# Honey Bee Genetic Stock Determines Deformed Wing Virus Symptom Severity but not Viral Load or Dissemination Following Pupal Exposure

**DOI:** 10.3389/fgene.2022.909392

**Published:** 2022-06-03

**Authors:** Hannah J. Penn, Michael D. Simone-Finstrom, Yanping Chen, Kristen B. Healy

**Affiliations:** ^1^ United States Department of Agriculture, Agricultural Research Service, Sugarcane Research Unit, Houma, LA, United States; ^2^ United States Department of Agriculture, Agricultural Research Service, Honey Bee Breeding, Genetics and Physiology Research Unit, Baton Rouge, LA, United States; ^3^ United States Department of Agriculture, Agricultural Research Service, Bee Research Laboratory, Beltsville, MD, United States; ^4^ Department of Entomology, Louisiana State University and AgCenter, Baton Rouge, LA, United States

**Keywords:** *Apis mellifera*, *Varroa destructor*, Pol-Line, Russian honey bees, genotype × genotype interactions, viral strain

## Abstract

Honey bees exposed to *Varroa* mites incur substantial physical damage in addition to potential exposure to vectored viruses such as Deformed wing virus (DWV) that exists as three master variants (DWV-A, DWV-B, and DWV-C) and recombinants. Although mite-resistant bees have been primarily bred to mitigate the impacts of *Varroa* mites, mite resistance may be associated with increased tolerance or resistance to the vectored viruses. The goal of our study is to determine if five honey bee stocks (Carniolan, Italian, Pol-Line, Russian, and Saskatraz) differ in their resistance or tolerance to DWV based on prior breeding for mite resistance. We injected white-eyed pupae with a sublethal dose (10^5^) of DWV or exposed them to mites and then evaluated DWV levels and dissemination and morphological symptoms upon adult emergence. While we found no evidence of DWV resistance across stocks (i.e., similar rates of viral replication and dissemination), we observed that some stocks exhibited reduced symptom severity suggestive of differential tolerance. However, DWV tolerance was not consistent across mite-resistant stocks as Russian bees were most tolerant, while Pol-Line exhibited the most severe symptoms. DWV variants A and B exhibited differential dissemination patterns that interacted significantly with the treatment group but not bee stock. Furthermore, elevated DWV-B levels reduced adult emergence time, while both DWV variants were associated with symptom likelihood and severity. These data indicate that the genetic differences underlying bee resistance to *Varroa* mites are not necessarily correlated with DWV tolerance and may interact differentially with DWV variants, highlighting the need for further work on mechanisms of tolerance and bee stock–specific physiological interactions with pathogen variants.

## 1 Introduction

Honey bee colony health is substantially impacted by the presence of the parasitic mite *Varroa destructor* (Anderson and Trueman), known to vector honey bee viruses such as Deformed wing virus (DWV) (Conte et al., 2010; Rosenkranz et al., 2010; Nazzi and Conte, 2016). Overt morphological symptoms of DWV, of which there are several prevailing variants (A, B, and C) and recombinants, include malformed wings and bloated abdomens (Moore et al., 2011; Zioni et al., 2011; Martin et al., 2012; Mordecai et al., 2016a; Mordecai et al., 2016b). Symptomatic adults die quickly upon emergence, while asymptomatic (i.e., no visible morphological changes) adults exhibit learning difficulties and diminished flight ability (Javaid and Uli, 2007; de Miranda and Genersch, 2010; Wells et al., 2016; Benaets et al., 2017). Such effects on individual bees may accumulate to impact whole colonies, with DWV infection decreasing the probability of colony survival (Berthoud et al., 2010; Dainat et al., 2012; Martin and Brettell, 2019).

One strategy to mitigate *Varroa* mite impacts on honey bee colony health and survival is breeding for mite resistance (Buchler, 1994; Harbo and Harris, 1999a; Rinderer et al., 2010; Mondet et al., 2020). Previous honey bee breeding programs have selected several parameters such as hygienic and grooming behaviors meant to decrease the mite load in the colony (Harbo and Harris, 1999b; Guzman-Novoa et al., 2012; Bąk and Wilde, 2015; Guichard et al., 2020; Mondet et al., 2020). Hygienic behavior, known to reduce the levels of diseases such as American and European foulbrood by the removal of damaged pupae, has also been shown to reduce *Varroa* mite loads in European honey bee colonies (Rothenbuhler, 1964; Spivak and Reuter, 2001a; Spivak and Reuter, 2001b). *Varroa-*sensitive hygienic bees (VSH) including the Pol-Line stock are a sub-selected group exhibiting hygienic behavior that targets mite-infested pupal cells rather than damaged pupae, removing reproductive *Varroa* mites, minimizing mite population growth (Harris, 2007; Rinderer et al., 2010; Danka et al., 2013; Danka et al., 2016; Mondet et al., 2016; Saelao et al., 2020; Spivak and Danka, 2021). Another mite-resistant stock, the Russian honey bee, was bred starting from a population historically associated with *Varroa* mites and was selected more generally for low mite population growth, which involves several mechanisms of defense such as brood breaking and heightened grooming (de Guzman et al., 2007; Rinderer et al., 2010). Similarly, the Saskatraz stock has also undergone selection primarily for survivorship and low mite population growth without targeting a single, specific mechanism (Robertson et al., 2014).

Furthermore, the genotypic differences underlying mite-resistant behaviors may be associated with reduced levels of mite-vectored viruses such as DWV (Locke et al., 2014; Khongphinitbunjong et al., 2016; de Guzman et al., 2017; Mendoza et al., 2020; Weaver et al., 2021; O’Shea-Wheller et al., 2022). Such bee genotype × DWV interactions may be the result of enhanced virus resistance or tolerance in certain bee stocks, with prior data indicating a greater potential for tolerance (Strauss et al., 2013; Locke et al., 2014; Khongphinitbunjong et al., 2015; Khongphinitbunjong et al., 2016; Thaduri et al., 2018; Thaduri et al., 2019; Locke et al., 2021). Resistance is the ability of the bee to prevent an infection from establishing or increasing after exposure, while tolerance is the ability of the individual to maintain health and functionality (i.e., reduced symptoms), even while having an infection (Locke et al., 2014; Mordecai et al., 2016a; Burgan et al., 2018). Host genotype × virus interactions may be direct through individual host immune responses to the virus or indirect *via* colony-level mite-removal traits (Chen and Siede, 2007; Khongphinitbunjong et al., 2015; de Guzman et al., 2019; Bouuaert et al., 2021; Weaver et al., 2021). For instance, genotype may mediate the bee’s ability to detect signals such as mite-related cuticular hydrocarbons; then, these detection differences can translate into differential hygienic responses resulting in cascading differences in mite and vectored virus levels (Mondet et al., 2021; Wagoner et al., 2019). In addition, virus levels may potentially feed back into this system by further disrupting the bee’s mite detection ability (Wagoner et al., 2019; Mondet et al., 2015). If direct mechanisms drive genotype-associated differences in virus levels, we expect to observe genotype differences in laboratory studies of virus loads as the need for mite-removal behavior is not necessary for virus reduction (Locke et al., 2014). Complicating this matter are potential differences among DWV variants, with conflicting reports indicating context-dependent variant virulence and likelihood of inducing symptoms or mortality (Mordecai et al., 2016a; McMahon et al., 2016; Brettell et al., 2017; Natsopoulou et al., 2017; Gisder et al., 2018; Barroso-Arévalo et al., 2019; Kevill et al., 2019; Tehel et al., 2019; Dubois et al., 2020; Norton et al., 2020).

In this study, we evaluated potential resistance or tolerance to DWV in five honey bee genetic stocks (Carniolan, Italian, Pol-Line, Russian, and Saskatraz), with varying levels of resistance to the *Varroa* mite using a laboratory environment to control for indirect behavioral mechanisms. The Italian and Carniolan stocks are commonly used throughout the commercial beekeeping industry and have been bred for honey production and colony size; whereas Russian, Pol-Line, and Saskatraz bees have been specifically bred for *Varroa* mite resistance (de Guzman et al., 2007; Robertson et al., 2014; Danka et al., 2016; Caron, 2021; O’Shea-Wheller et al., 2022). These genetic stock differences represent different genotypes in a broad sense as they have clear differences in phenotype and are derived from different breeding populations. The specific genotypic information was not further assessed as part of this study though it can be found, in part, in prior studies (Jiang et al., 2016; Wang, 2016; Saelao et al., 2020). To determine potential interactions of bee stock and DWV, we exposed honey bee pupae, the life stage that is most commonly infested with mites (Ifantidis, 1983, 1984; Donzé and Guerin, 1994), to *Varroa* mites or injected them with a sublethal dose (10^5^) of DWV sourced from symptomatic (e.g., deformed wings) adult bees. Upon adult emergence, we determined DWV levels and dissemination throughout different tissue types [abdomen, head, hypopharyngeal gland, and a rear leg as in the study by Penn et al. (2021)], the number of days until adult emergence, and DWV symptom presence and severity. The tissue types selected were chosen as legs have been used to indicate viral dissemination in other arthropods (Boncristiani et al., 2009; Diagne et al., 2015); the head has been an indicator of overt bee infections (Yue and Genersch, 2005; Möckel et al., 2011); hypopharyngeal glands may provide possible transmission by food trophallaxis (Chen Y. et al., 2006, Chen et al., 2006 Y. P.; Möckel et al., 2011); and the abdomen as the site of mite feeding and our injection treatment groups. Since there are two common variants (A and B) of DWV in the United States and the potential virulence and mortality effects may differ based on interactions between host and viral genotypes (Dainat et al., 2012; Ryabov et al., 2017; Kevill et al., 2019; Loope et al., 2019; Grindrod et al., 2021), we tested for RNA copy levels of both DWV-A and DWV-B variants.

## 2 Materials and Methods

### 2.1 Source Colonies

All honey bee colonies were started from 0.90 to 1.10 kg “packages” made on 3 May 2018 from 10 established colonies of the USDA Honey Bee Breeding, Genetics and Physiology Research Unit in Baton Rouge, LA, United States (30°22′56″ N, 91°10′40″ W). Naturally mated queens from five genetic stocks were sourced from the USDA laboratory (Pol-Line and Russian), a Canadian collaborating breeder (Saskatraz), or purchased from commercial California suppliers (Carniolan and Italian). Queens were queen candy released into colonies on 4 May 2018. Colonies (*N* = 3 colonies/stock) were not sampled until 6 weeks post queen introduction and supplementation to allow time for population turnover to reflect queen genetics. All colonies were similarly maintained in three yards near the USDA laboratory (with Carniolan, Italian, and Saskatraz sharing one yard, while Pol-Line and Russian colonies were maintained in two separate yards). To allow for direct comparison, the same colonies were used in a complementary study following DWV levels over 10 days in newly emerged adult bees (Penn et al., 2021).

### 2.2 Viral Isolation and Mite Sourcing

To obtain the DWV viral solution for injection, 20 adult honey bees with overt DWV symptoms were frozen at −80°C, ground to a fine powder, homogenized in 10 ml 1X PBS, and centrifuged at 5,000 rpm for at 4°C for 20 min. The resulting supernatant containing viruses was filtered through a 0.2-micron filter (milex-GS syringe filter unit #SLGS033SS, Millipore Sigma, Burlington, MA, United States) to remove small tissue debris, fungi, and bacteria. qPCR was conducted to test for non-target viruses (acute bee paralysis virus, black queen cell virus, chronic bee paralysis virus, Israeli acute paralysis virus, Kashmir bee virus, and Lake Sinai virus) using the methods mentioned later. DWV quantification using general DWV (not variant-specific) primers (Supplementary Table S1) was performed by absolute quantification using the standard curve method. All methods were previously established based on standard protocols (Simone-Finstrom et al., 2018). One sample stock solution was selected based on negative results for non-target viruses and used to create the injection stock solution. The stock solution was diluted to 10^5^ viral copies of DWV, a biologically relevant but sublethal to adult bees (Gisder et al., 2009). For the mite inoculation treatment group, *Varroa* mites were obtained from non-study hives using powdered sugar rolls to dislodge live mites from nurse bees (Macedo et al., 2002). Dislodged mites were removed from powdered sugar using a paint brush and stored in a petri dish containing a maximum of 100 mites, allowed to feed on 10 previously un-infested bee pupae (exchanged daily), and maintained in the incubator at 34°C and 85% relative humidity until use within 24–48 h (Dietemann et al., 2013).

### 2.3 Pupal Assay

From mid-July to early October 2018, frames with white-eyed pupae were removed from each colony and brought back to the laboratory where the *Varroa* mite-free (no mites in pupal cells or on body) pupae were removed, placed on a folded filter paper in petri dishes, and stored in an incubator at 34°C and 85% relative humidity. Pupae exhibiting any discoloration due to damage during handling after 2 h were removed from the experiment, while healthy pupae (17 treatment/colony) were assigned to one of four treatment groups—1) no manipulation (included since bees had naturally occurring DWV infections, referred to as “control”), 2) 3.0 µl 1X PBS injection to control for injection damage (PBS), 3) 3.0 µl 10^5^ DWV injection to simulate the vectoring of DWV without mite presence (DWV), or 4) *Varroa* mite inoculation (mite). Control bees were placed into an individual, size “1” gel capsules (Capsule Connection, Prescott, AZ, United States), with a small hole in the top created using an insect pin (Nazzi and Milani, 1994; Piou et al., 2016; Posada-Florez et al., 2019). All pupae in the PBS and DWV treatment groups were injected using an UltraMicroPump with an SYS-Micro4 controller (World Precision Instruments, Sarasota, FL, United States) with an infusion flow rate of 1.0 μl/s, following the manufacturer’s parameters. For each injection, a 30G needle (Hamilton Company, Reno, NV, United States) was inserted into the lateral abdomen between the fourth and fifth pleurites, based on established protocols (Simone-Finstrom et al., 2018). After injection, pupae were then transferred to individual gel capsules as mentioned earlier. For the mite treatment group, pupae were transferred to an individual gel capsule followed by an individual mite (Dietemann et al., 2013).

**FIGURE 1:**
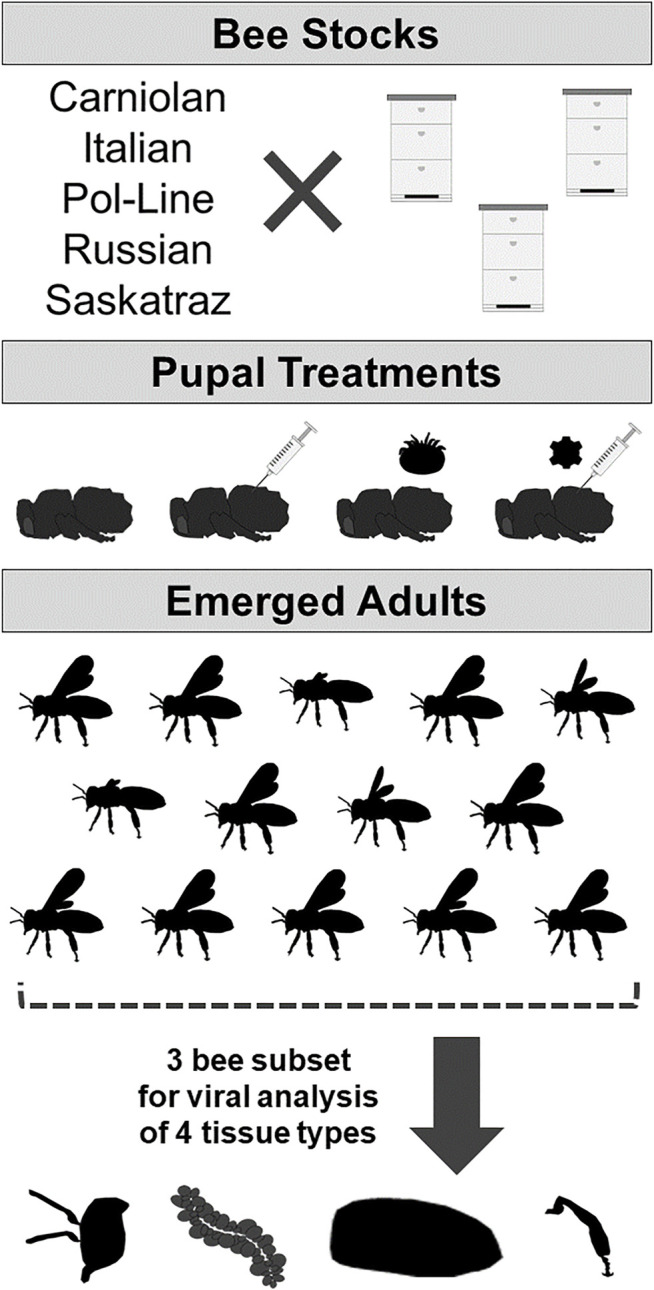
Experimental design: experimental design to determine DWV symptoms and levels based on bee stock and treatment of white-eyed pupae (no injection control/naturally occurring infection, PBS sham injection, *Varroa* mite exposure, or sublethal DWV injection). DWV symptoms were analyzed in emerged adults (N = 12–17 bees per stock/treatment combination); then a subset of three bees was dissected into four tissue types (heads, hypopharyngeal glands, abdomen, and rear legs) subsequently used for viral analyses.

All pupae were incubated at 34°C and 85% relative humidity until adult emergence, with all bees checked daily and necrotic individuals removed. Mite treatments where the mite was dead or did not defecate (an indication that no feeding occurred) by adult bee emergence were not used in subsequent analysis but were replaced with replicates meeting the requirements. Upon emergence, all adult bees were evaluated for DWV symptoms and rated on a scale of severity from 0 to 3 (Supplementary Figure S1), with 0 indicating normal wings, 1 indicating slight malformation, 2 indicating major malformations but with wings present, and 3 indicating completely malformed. Mites were removed from respective emerged bees; then all mites and bees were placed into individual sterile 1.5-ml centrifuge tubes and stored at −80°C. The first emerging bees per colony per stock per treatment group combination were evaluated in this manner (*n* = 12–17 bees/colony/treatment group/stock, N = 981 total emerged bees) and then a random subset of three bees was selected for tissue dissection and DWV quantification (Figure 1).

### 2.4 Adult Assay

The adult assay was part of a complementary study on DWV dissemination in injected adult bees over time (Penn et al., 2021). The same colonies were used for the adult injection as the pupal injection experiment and throughout the same timeframe (July through October 2018). *Varroa* mite–free newly emerged bees were treated similarly, except for the no mite treatment group, and all bees were placed on ice for 2 m prior to injection to reduce movement. Bees were injected using the same methods as aforementioned with a 3.0 µl of DWV inoculum from an aliquot of the same inoculum as used for the pupae experiment (DWV) or 3 µl 1X PBS (PBS) or had no injection (control) were implemented as controls. The bees were housed in cages of maximum 30 bees and provided with 50% sucrose solution and pollen substitute. The bees were maintained in an incubator at 34°C and 85% relative humidity and then sacrificed at 1, 2, 4, 7, and 10 days post injection. Only day seven data (N = 135 bees and 540 associated RNA extractions) were included within this study as 7 days allows for a similar period of viral replication compared to pupae data.

### 2.5 Dissection and RNA Isolation

To determine virus dissemination through the bee body over time, the randomly selected three-bee subset from each colony/treatment group/stock combination was dissected in the same manner as the complementary study on adult bees (Penn et al., 2021). Dissections were conducted over dry ice with individuals dissected with a new, sterilized blade. The body was separated into legs, wings, head, thorax, and abdomen; the head was embedded into beeswax (new per individual bee) and the hypopharyngeal gland was removed according to the previously published methods (Corby-Harris and Snyder, 2018; Penn et al., 2021). Dissected tissues were stored in separate sterilized tubes on dry ice during dissection and long term at −80°C.

Total RNA was extracted for a single rear leg, the head (sans hypopharyngeal gland), hypopharyngeal gland, and abdomen for each of the three bees representing each combination of colony/treatment group/stock (N = 180 bees total and N = 720 RNA extractions in total) and all mites (N = 45). Individual hypopharyngeal glands and mites were placed in 30 µl lysis buffer and 30 μl Maxwell homogenization buffer and vortexed. The leg (cut into pieces), head, and abdomen were placed in 200 µl lysis buffer and 200 μl Maxwell homogenization buffer (Promega Corporation, Madison, Wisconsin, United States), manually ground with a pestle (Sigma-Aldrich, St. Louis, Missouri, United States), and vortexed. All samples were then incubated for 90 min at 4°C. After incubation, 320 μl Maxwell homogenization buffer was added to the hypopharyngeal gland and mite samples. The samples were then extracted using the Maxwell RSC 48 cartridges (Promega Corporation, Madison, Wisconsin, United States). The total RNA was extracted according to standard procedures using RSC simplyRNA tissue extraction kits and program (Promega Corporation, Madison, Wisconsin, United States). RNA was stored in 0.6-ml elution tubes wrapped in parafilm (Bemis NA, Neenah, Wisconsin, United States) at −80°C.

### 2.6 cDNA Synthesis and RT-PCR

Frozen RNA samples were thawed on −20°C metal beads, briefly vortexed, and then centrifuged. Each RNA sample was nano-dropped (NanoDrop One, Thermo-Fisher Scientific Inc., Waltham, Massachusetts, United States) twice using 1 μl of sample. The mean ng/µl NanoDrop One readings (for 260/280 and 260/230) were calculated per sample and then used to determine adequate sample purity and the quantities of sample and nuclease-free water required to dilute each sample to a concentration of 250 ng of RNA. cDNA was then synthesized in two steps using Qiagen QuantiTect Reverse Transcription kits (Thermo-Fisher Scientific Inc., Waltham, Massachusetts, United States). For step 1, 2 µl of gDNA wipeout buffer was added to the mix of RNA and water for a total reaction volume of 14 µl per sample. The samples were incubated at 42°C for 2 min in a Bio-Rad T100 Thermal Cycler (Bio-Rad, Hercules, California, United States). The samples were then briefly vortexed and centrifuged before the addition of 4 µl 5X buffer, 1 µl of RT primer mix, and 1 µl of RT enzyme per sample. The samples were again vortexed and centrifuged and then placed into the Bio-Rad T100 Thermal Cycler (42°C for 25 min then 95°C for 3 min). cDNA was stored in strip tubes wrapped in parafilm at −80°C.

All samples were tested with DWV-A and DWV-B primers using qRT-PCR to determine infection levels (primers in Supplementary Table S1), and each sample was replicated two times per primer pair. All qRT-PCRs consisted of 5 µl SsoFast Universal SYBR Green Supermix (Bio-Rad, Hercules, California, United States), 3 µl nuclease-free water, 0.5 µl forward primer, 0.5 µl reverse primer, and 1 µl cDNA from each sample. All reactions were run in Bio-Rad CFC 96 or Connect thermal cyclers (Bio-Rad, Hercules, California, United States), with all reactions of a specific primer pair occurring in the same machine. The PCR cycling protocol for DWV-A was 95°C for 1 min followed by 40 cycles of 95°C for 10°s and 60°C for 15 s then 65°C for 5°s; while the protocol for DWV-B was 95°C for 5 min followed by 40 cycles of 95°C for 5°s and 52.5°C for 10 s then 72°C for 10 s. The thermal protocols included a melt-curve dissociation analysis to confirm the product size. DWV-A and DWV-B results were quantified using the standard curve method using linearized plasmid constructs (ranging from 10^5^–10^12^). Quantified virus RNA copy levels (DWV RNA equivalents per 10 ng of RNA) were log-transformed for analyses.

### 2.7 Statistical Analyses

All statistical analyses were conducted in R 4.0.2 (R Core Team, 2020), and all graphs were plotted using ggplot2 (Wickam, 2016). To determine factors influencing DWV-A and DWV-B levels and dissemination to different tissue types, the three bee sub-samples with virus data were used with all tissue types included in the analyses. Separate general linear mixed models (GLMMs) were generated for each DWV variant (DWV-A and B) using the lme4 (glmer function with Gaussian distribution) package (Bates et al., 2015). Variables for model selection included bee stock, treatment group, tissue type, all interaction terms of those three variables, and the levels of the alternate DWV variant. Colony and individual bees were used as random effects to account for multiple tissue types coming from the same individual and individuals from the same colony. The Italian bee stock, control treatment group, and head tissue were specified as model intercept values. Backward model selection was conducted using a combination of minimum AICc and BIC values; models with significantly lower scores (Δ > 4) were used. It is to be noted that the stock × treatment group interaction term was tested in model selection and was neither significant nor did it contribute to model fit so was not included in the final model. Final model information can be found in Supplementary Table S2. Significance values were estimated using Satterthwaite approximation with the lmerTest package (Kuznetsova et al., 2017). *Post hoc* comparisons were conducted with Kenward–Roger estimation in the emmeans package (Lenth, 2020).

This experiment also allowed us the unique opportunity to evaluate DWV levels and dissemination differences between this experiment and prior work with injected newly emerged adults from the same colonies at the same time of the same year of collection (Penn et al., 2021). To determine if differences occurred between these two experiments, the mite treatment group was dropped from this pupae experiment as it was not included as a treatment group in the adult experiment. Similarly, we only evaluated the day 7 time-point from the adult experiment as that matched the pupae experiment in terms of number of days post injection. Day 7 comparison models were conducted as aforementioned except that experiment and associated interaction terms were included in the model and the adult experiment was used in the model intercept.

The time (days) until adult emergence was modeled using a GLMM using the lme4 (glmer function with Gaussian distribution) package (Bates et al., 2015). To avoid redundant sampling per bee, only head tissue (sans hypopharyngeal glands) data were used as this particular tissue type was the best representation of infections for both DWV variants (Schurr et al., 2019; Dubois et al., 2020; Penn et al., 2021). Variables considered for model selection included bee stock, treatment group, stock × treatment group, and DWV-A and -B levels; colony was used as a random effect. The Italian bee stock and the control treatment group were specified as model intercept values. Model selection and *post hoc* comparisons were conducted as mentioned earlier. The presence of DWV symptoms was analyzed similarly using a GLMM using the lme4 (glmer function with binomial distribution) package but with a logit link function within a binomial logistic regression (presence/absence of symptoms).

DWV symptom severity (scale of 0–3) was modeled using an ordered logistic regression using the polr function (logistic) in MASS (Ripley et al., 2018). Marginal effects and predicted probabilities for the treatment group and stock comparisons were calculated using the effects package (Fox, 2003; Fox and Hong, 2009; Fox and Weisberg, 2019). Variables and methods for model selection were as mentioned previously (time to emergence and symptom presence). We further synthesized the results of the symptom presence and severity models using classification trees. The two-response categorical variable for DWV symptom presence (0, 1) and the four-response ordinal variable for DWV symptom severity (wind deformity on a scale of 0–3) were used to construct two classification trees with the same predictor variables as the associated logistic models. The classification trees were created and plotted using recursive partitioning provided in the rpart and rpart plot packages (Milborrow, 2017; Therneau et al., 2018). All predictor variables were maintained in the final classification trees with each having a complexity parameter >0.01 after pruning. The relationships among bee stock, treatment group, DWV levels, and the DWV symptom severity scale were further modeled using multiple correspondence analysis (MCA) and factor analysis of mixed data (FAMD) using the FactoMineR package (Husson et al., 2008). MCA allows for the analysis of multiple qualitative variables (similar to PCA with quantitative data), whereas FAMD analyzes the relationships between the combinations of qualitative and quantitative variables. Variables presented closer together on the plot are more similar to each other than those further away (like on the opposite side of the origin).

**FIGURE 2:**
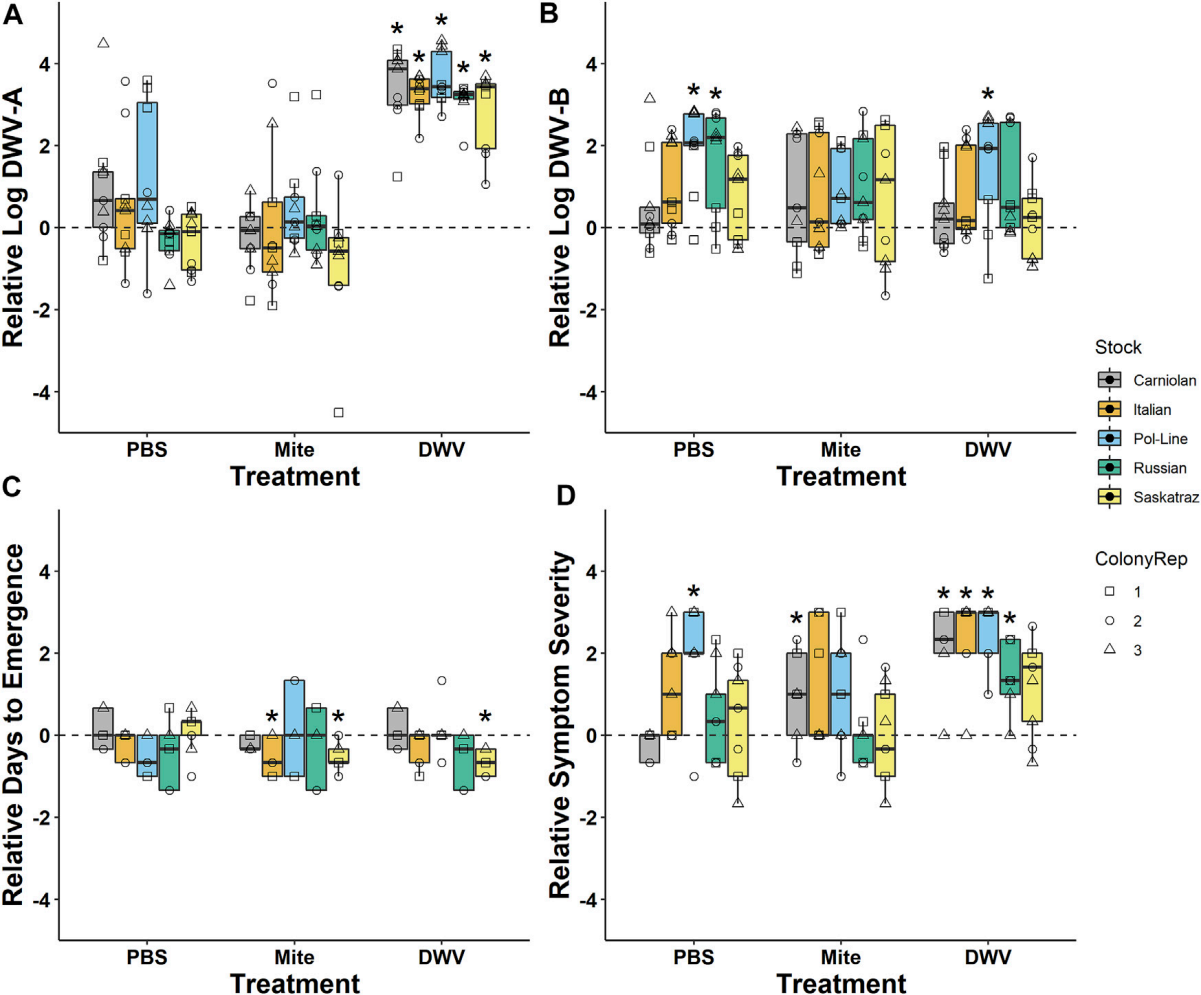
Treatment values relative to naturally occurring levels. Comparison of **(A)** log-transformed DWV-A and **(B)** DWV-B levels in head tissues indicates that the pupae DWV injection treatment sourced from symptomatic adults successfully increased DWV-A but not DWV-B levels in all bee stocks. **(C)** Treatment × bee stock interactions decreased the number of days from pupal treatment until adult emergence compared to the control. **(D)** DWV symptom severity generally increased in the DWV treatment but interacted with bee stock. Values were made relative to the untreated control bees from the same colony with naturally occurring infections (y = 0). Boxplots are in the style of Tukey where the box limits represent the lower 25% quantile and upper 75% quantile with the line representing the median. Individual points indicate individual bees (N = 9 bees/treatment/stock) with shapes associated with colony replicate (ColonyRep) and color indicating stock. * Denotes significant differences between the treatment and control values (Dunnett’s test; *p* < 0.05).

For ease of graphical representation, PBS, mite, and DWV pupae treatment groups were also compared against the control treatment group with regards to log-transformed DWV-A and DWV-B levels, days until emergence, and symptom severity. This was performed by calculating the mean for the control data for each of the three replicates per tissue type, treatment group, and colony. The mean control value was assumed to be the baseline (y = 0 value on Figure 2) value for that colony × tissue type × treatment group combination and was then subtracted from each of the three replicates per treatment group for that colony and tissue type. Statistical significance represented in Figure 2 was determined by using *post hoc* comparisons for the treatment group, described earlier.

**FIGURE 3:**
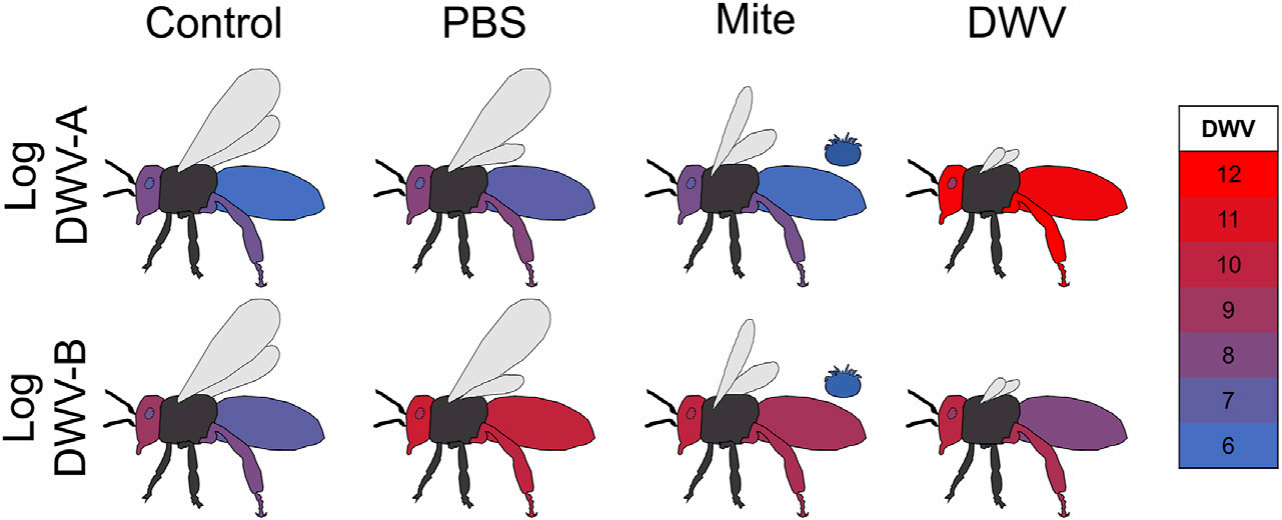
DWV dissemination in newly emerged adult bees. Mean log-transformed DWV-A and DWV-B values (color values scaled for overall DWV levels) in each sampled tissue type (abdomen, head, hypopharyngeal gland, and rear leg) for each treatment (uninjected control, PBS injection, *Varroa* mite exposure, and DWV injection). Dissemination patterns differed between the two DWV variants but was not related to bee stock (displayed DWV values were averaged across bee stocks).

**FIGURE 4:**
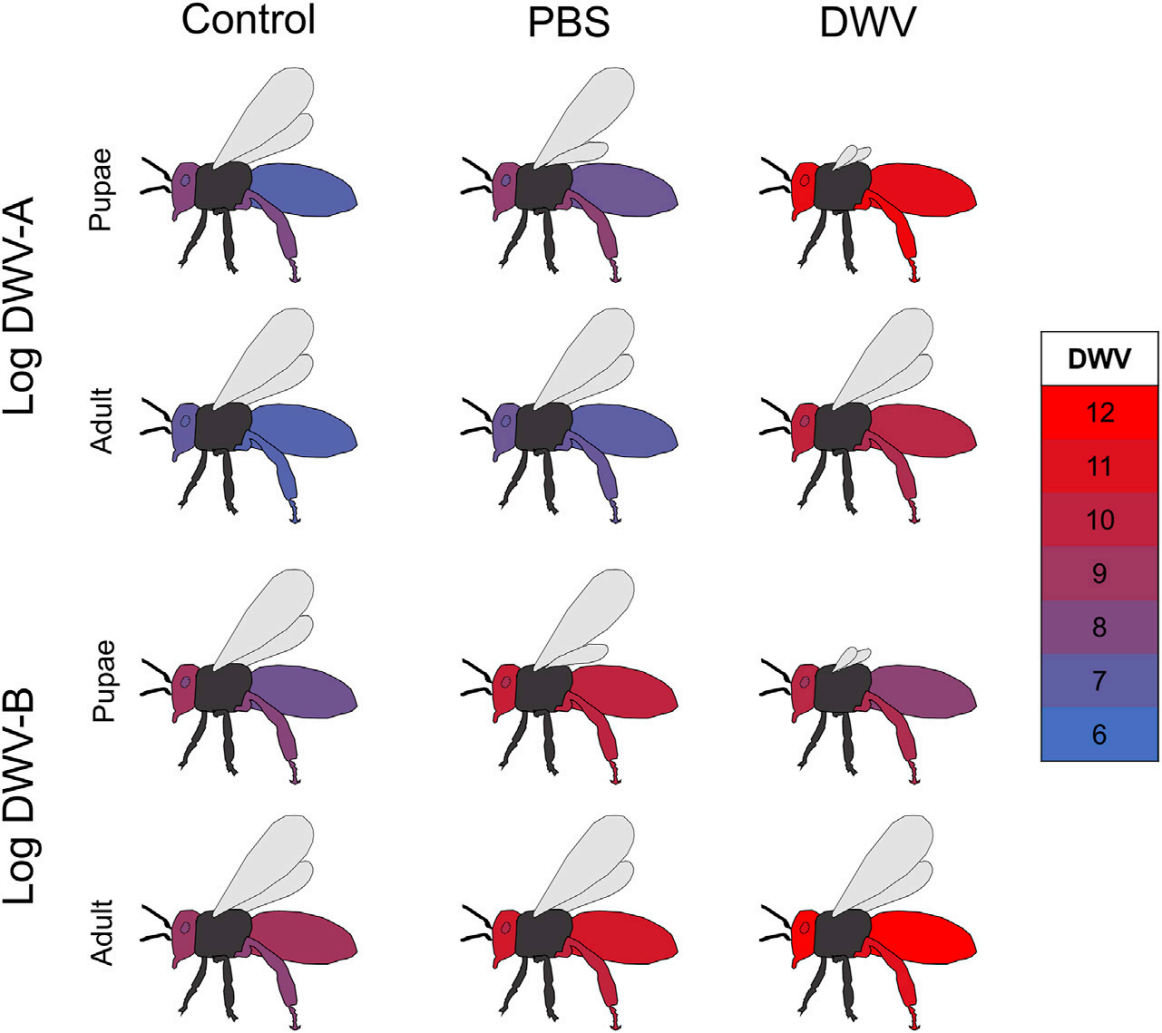
Comparison of DWV dissemination in treated pupae and adults. Mean log-transformed DWV-A and DWV-B values (color values scaled for overall DWV levels) in each sampled tissue type (abdomen, head, hypopharyngeal gland, and rear leg) for each treatment (uninjected control, PBS injection, and DWV injection) overlapping between treated pupae and treated newly emerged adult experiments. The pupae experiment represents data described in this manuscript (bees injected as white-eyed pupae but sampled upon adult emergence about 7–8 days post-treatment), while the adult data represent samples of injected newly emerged adults collected 7 days post treatment Supplementary Figure S2 for the comparison of DWV for the combination of stock, treatment, and experiment.

Graphical representation of DWV dissemination (Figures 3, 4) was conducted in Microsoft 365 Excel and PowerPoint. The mean log-transformed DWV-A and DWV-B values (across all bee stocks) per treatment group were calculated and copied and pasted into a column in an Excel spreadsheet with an associated treatment group and variant labeling in the prior column. Whole numbers (as represented by the color scale in Figures 3, 4) were placed through this column and encompassed the minimum and maximum values of both the DWV-A and DWV-B values. The DWV values were then selected and colored using the conditional formatting tool where the lowest whole number was assigned blue (RGB: 0, 112, 192; Hex #0070C0) and the largest whole number was assigned red (RGB: 255, 0, 0; Hex #FF0000). This color scale and precise values based on the tissue type × treatment group means were pasted into PowerPoint as an image to maintain the appropriate color scale. Graphical outlines of bees were generated by the authors in PowerPoint, and each tissue type per treatment group was colored using the corresponding DWV values with the eyedropper tool.

## 3 Results

### 3.1 Deformed Wing Virus Levels and Dissemination

#### 3.1.1 Treatment Impacts on Viral Load

When we analyzed DWV levels in the newly emerged adult bees, we found that the treatment group was significant but differed with the DWV variant (Table 1; Supplementary Table S3). Overall, DWV-A levels were higher in the DWV-injected bees than in the untreated control bees with naturally occurring infection (Kenward–Roger estimation, *t =* 9.279, *p* < 0.001), PBS (*t =* 6.061, *p* < 0.001), or mite treatments (*t =* 7.595, *p* < 0.001) (Figure 2). Unlike DWV-A, PBS injection and mite infestation resulted in higher DWV-B levels than those of the untreated bees (Kenward–Roger estimation, compared to PBS: *t* = 5.208, *p* < 0.001; compared to mite: *t =* 4.463, *p* < 0.001) and the DWV injection treatment group (compared to PBS: *t* = 3.218, *p* = 0.008; compared to mite: *t =* 2.489, *p* = 0.065). Moreover, the DWV treatment group did not differ in DWV-B levels from the untreated control group (Kenward-Roger estimation, *t =* 1.664, *p* = 0.346). Mites used for the infestation treatment group had significantly lower levels of DWV-A (10^2.42 ± 0.34^) than the DWV-B level (10^4.82 ± 0.20^) (ANOVA, F_(1, 87)_ = 36.735, *p* < 0.001), but mites expressed similar levels of each variant regardless of bee stock that they were infesting (DWV-A: ANOVA, F_(4, 43)_ = 0.642, *p* = 0.636; DWV-B: ANOVA, F_(4, 43)_ = 0.556, *p* = 0.696).

**TABLE 1:** DWV dissemination ANOVAs. ANOVA summary of the generalized linear mixed-model fixed effects for the log-transformed DWV-A and DWV-B RNA copy levels disseminated throughout the newly emerged adult tissues (N = 180 bees total and N = 720 RNA extractions in total). Supplementary Table S1 for model parameter estimates.

Variable	Log DWV-A						Log DWV-B					
	SumSq	MeanSq	NumDF	DenDF	*F*-value	*p*-value	SumSq	MeanSq	NumDF	DenDF	*F*-value	*p*-value
Stock	1.31	0.33	4	10.01	0.25	0.902	3.42	0.85	4	9.96	0.81	0.546
Treatment	385.44	128.48	3	160.11	98.99	0.000	36.42	12.14	3	179.71	11.52	0.000
Tissue type	137.26	45.75	3	468.80	35.25	0.000	66.08	22.03	3	462.19	20.91	0.000
Treatment*tissue	102.05	11.34	9	457.46	8.74	0.000	44.05	4.89	9	457.43	4.65	0.000
Log DWV-A							11.31	11.31	1	565.07	10.74	0.001
Log DWV-B	5.23	5.23	1	516.10	4.03	0.045						

#### 3.1.2 Deformed Wing Virus Levels in Different Tissues

Bee stock did not significantly impact overall DWV-A levels (Table 1; Supplementary Table S3; Figure 2), indicating that genetic stocks are not resistant to DWV, given the exposure circumstances of this experiment. However, both DWV variants varied significantly but differentially among the tissue types (Table 1; Supplementary Table S3; Figure 3). Pooling all treatment groups, for DWV-A, heads and legs had higher levels than abdomens (Kenward–Roger estimation, compared to heads: *t = −*7.224, *p* < 0.001; compared to legs: *t = −*9.279, *p* < 0.001) and hypopharyngeal glands (compared to heads: *t = −*7.250, *p* < 0.001; compared to legs: *t = −*7.406, *p* < 0.001). With DWV-B levels, heads had higher levels than all other sampled tissues (compared to abdomen: *t* = 7.750, *p* < 0.001; compared to hypopharyngeal gland: *t* = 4.756, *p* < 0.001; compared to leg: *t =* 4.832, *p* < 0.001), and legs had higher levels than abdomens (*t =* 3.325, *p* = 0.005) but were not different from hypopharyngeal glands (*t =* 0.784, *p* = 0.862). Overall, heads and legs had the highest viral loads of both DWV genotypes.

Tissue type significantly interacted with the treatment group for both DWV-A and B levels (Table 1; Supplementary Table S3; Figure 3). Within the control treatment group, heads, legs, and hypopharyngeal glands had similar levels of DWV-A; but heads and legs exhibited higher DWV-A levels than those of the abdomens (Table 2). For DWV-B in the control treatment group, heads had the greatest virus levels and legs had higher levels than those of abdomens but were not different from hypopharyngeal glands (Table 2). In the PBS treatment group, both heads and legs had higher DWV-A levels than abdomens and hypopharyngeal glands (Table 2). Abdomens had lower DWV-A levels than all other tissue types in the mite treatment group (Table 2). For DWV-B in the PBS treatment group, the only significant difference was between heads and hypopharyngeal glands; and there were no observed differences in DWV-B levels among tissue types in the mite treatment group (Table 2). In the DWV injection treatment group, hypopharyngeal glands had lower DWV-A levels than all other tissue types (Table 2). For DWV-B levels in the DWV treatment group, abdomens had lower levels than all other dissection components levels, which were not different from each other (Table 2).

**TABLE 2:** Tissue-type contrasts. *Post hoc* contrasts comparing DWV-A (A) and DWV-B (B) levels among tissue types [head, hypopharyngeal gland (hyp. gland), rear leg (leg), and abdomen] for each treatment [naturally occurring infection (control), PBS injection, DWV injection and *Varroa* mite exposure (mite)]. Statistical significance is also indicated by “*”.

Treatment	Tissue type	Head	Hyp. gland	Leg	Abdomen
Control	Head		A: *t* = 2.183, *p* = 0.129	A: *t* = 0.769, *p* = 0.869	A: *t* = 5.221, *p* < 0.001*
	Hyp. gland	B: *t* = 4.184, *p* < 0.001*		A: *t* = −1.538, *p* = 0.416	A: *t* = −2.486, *p* = 0.063
	Leg	B: *t* = 3.961, *p* < 0.001*	B: *t* = −0.803, *p* = 0.853		A: *t* = −4.590, *p* < 0.001*
	Abdomen	B: *t* = 6.418, *p* < 0.001*	B: *t* = −1.543, *p* = 0.413	B: *t* = −2.662, *p* = 0.040	
PBS	Head		A: *t* = 1.014, *p* = 0.741	A: *t* = −0.069, *p* = 0.999	A: *t* = 4.919, *p* < 0.001*
	Hyp. gland	B: *t* = 2.951, *p* = 0.017*		A: *t* = −1.075, *p* = 0.705	A: *t* = −3.316, *p* = 0.005*
	Leg	B: *t* = 1.543, *p* = 0.413	B: *t* = −1.647, *p* = 0.354		A: *t* = −5.011, *p* < 0.001*
	Abdomen	B: *t* = 0.776, *p* = 0.865	B: *t* = 2.183, *p* = 0.129	B: *t* = 0.656, *p* = 0.913	
DWV	Head		A: *t* = 3.823, *p* < 0.001*	A: *t* = 0.273, *p* = 0.993	A: *t* = 4.806, *p* < 0.001*
	Hyp. gland	B: *t* = 2.142, *p* = 0.141		A: *t* = −3.264, *p* = 0.002*	A: *t* = −0.573, *p* = 0.940
	Leg	B: *t* = 1.945, *p* = 0.211	B: *t* = −0.536, *p* = 0.950		A: *t* = −4.584, *p* < 0.001*
	Abdomen	B: *t* = 7.029, *p* < 0.001*	B: *t* = −3.699, *p* = 0.001*	B: *t* = −5.105, *p* < 0.001*	
Mite	Head		A: *t* = −1.873, *p* = 0.241	A: *t* = 4.352, *p* < 0.001*	A: *t* = 4.700, *p* < 0.001*
	Hyp. gland	B: *t* = 0.521, *p* = 0.954		A: *t* = 4.181, *p* < 0.001*	A: *t* = −3.787, *p* = 0.001*
	Leg	B: *t* = 2.218, *p* = 0.120	B: *t* = −1.407, *p* = 0.496		A: *t* = 0.994, *p* = 0.753
	Abdomen	B: *t* = 2.105, *p* = 0.153	B: *t* = −1.341, *p* = 0.537	B: *t* = 0.043, *p* = 0.999	

#### 3.1.3 Deformed Wing Virus Levels in Injected Pupae and Adults After 7 Days

In order to compare DWV levels and dissemination differences based on the life stage (pupae or newly emerged adult) at the time of treatment, we modeled DWV-A and DWV-B levels for this experiment in conjunction with data from a complementary study on adult bees (Penn et al., 2021). While different cohorts of bees were used within each colony for the pupae and adult studies, the sampling of these cohorts occurred within the same months. In the adult bee study, newly emerged bees from the same colonies and time of year were injected with different aliquots of DWV from the same inoculum at the same concentration. Comparisons of data from the newly emerged bees treated as pupae in the current study were made to the data from 7 days post injection in the adult study as this timeline allows for a similar period of viral replication between the two datasets. We found that for both DWV-A and DWV-B, the experiment (adult vs. pupae injection) was significant, interacted with the treatment group and tissue type individually (Figure 4; Supplementary Figure S2), and interacted with the treatment group and tissue type in a three-way interaction (Table 3). Within each treatment group, pupae had significantly greater levels of DWV-A than adults (Kenward–Roger estimation, control: *t = −*2.921, *p* = 0.004; PBS: *t = −*3.151, *p* = 0.002; DWV: *t = −*4.760, *p* < 0.001). However, this differed for DWV-B levels, where adults had higher amounts than pupae within all treatment groups but was only significant in the DWV treatment group (control: *t* = 1.795, *p* = 0.074; PBS: *t* = 1.349, *p* = 0.179; DWV: *t =* 8.150, *p* < 0.001). The experiment also exhibited a three-way interaction with bee stock and treatment for DWV-A but did not interact with bee stock for DWV-B (Table 3).

**TABLE 3:** Day 7 comparison ANOVAs. ANOVA summaries for log DWV-A and DWV-B linear mixed models analyzing day 7 values for pupal and adult-injected bees (experiment). For pupal injections, the mite treatment was excluded; all other treatments remained in the analysis (control, PBS injection, and sublethal DWV injection). Each experiment was conducted on the same three colonies of each of the five stocks with the same four tissue types analyzed.

Variable	Log DWV-A						Log DWV-B					
	SumSq	MeanSq	NumDF	DenDF	*F*-value	*p*-value	SumSq	MeanSq	NumDF	DenDF	*F*-value	*p*-value
Stock	1.04	0.26	4	10.01	0.27	0.891	9.29	2.32	4	10.01	2.26	0.134
Treatment	359.39	179.69	2	232.63	185.90	0.000	104.20	52.10	2	232.61	50.78	0.000
Tissue type	122.20	40.73	3	733.67	42.14	0.000	82.51	27.50	3	694.18	26.81	0.000
Experiment	37.80	37.80	1	232.63	39.11	0.000	43.66	43.66	1	232.62	42.55	0.000
Stock*treatment	28.16	3.52	8	230.80	3.64	0.001	4.63	0.58	8	230.01	0.56	0.807
Stock*tissue	11.70	0.98	12	731.74	1.01	0.439	49.67	4.14	12	692.16	4.03	0.000
Stock*experiment	6.44	1.61	4	231.53	1.67	0.159	2.59	0.65	4	231.56	0.63	0.640
Treatment*tissue	74.75	12.46	6	733.66	12.89	0.000	13.39	2.23	6	694.32	2.18	0.044
Treatment*experiment	1.93	0.97	2	232.63	1.00	0.370	29.74	14.87	2	232.61	14.49	0.000
Tissue*experiment	56.15	18.72	3	733.69	19.36	0.000	77.73	25.91	3	694.17	25.25	0.000
Stock*treatment*experiment	23.85	2.98	8	230.80	3.08	0.003	14.30	1.79	8	230.02	1.74	0.090
Treatment* tissue*experiment	19.88	3.31	6	733.66	3.43	0.002	21.16	3.53	6	694.33	3.44	0.002

### 3.2 Time Until Adult Emergence

After treatment, pupae were checked every 24 h for adult emergence. The time (days) until adult emergence was impacted by the treatment group, stock × treatment group interactions, and DWV-B levels (Tables 4, 5) and was monitored. In the model, the mite treatment group was significantly negatively associated with the number of days until emergence (Table 5; Supplementary Table S4). Pol-Line bees assigned to the mite treatment group took less than half a day longer to emerge than Italian (Kenward–Roger estimation, *t =* 3.332, *p* = 0.022), Carniolan bees (*t =* 3.227, *p* = 0.027), and Saskatraz (*t =* 2.822, *p* = 0.065) bees exposed to mites (Table 5; Supplementary Table S4). In contrast, higher DWV-B levels decreased the number of days until adult emergence (Table 5; Supplementary Table S4), so bees with higher DWV-B developed more quickly.

**TABLE 4:** Mean days to emergence. The mean number of days until adult emergence (± standard error of the mean) for all observed bees and the nine-bee subset (per stock and treatment combination) used for viral analyses.

Stock	Treatment				Avg. Stock
	*Control*	*PBS*	*Mite*	*DWV*	
*Carniolan*					
All bees	7.88 ± 0.0a8	7.94 ± 0.09	7.59 ± 0.07	7.49 ± 0.08	7.73 ± 0.04
Subset	7.22 ± 0.15	7.33 ± 0.17	7.00 ± 0.00	7.33 ± 0.17	7.22 ± 0.07
*Italian*					
All bees	8.06 ± 0.09	7.75 ± 0.10	7.34 ± 0.07	7.27 ± 0.06	7.61 ± 0.05
Subset	7.56 ± 0.18	7.33 ± 0.17	7.00 ± 0.00	7.22 ± 0.15	7.28 ± 0.08
*Pol-Line*					
All bees	8.47 ± 0.11	7.73 ± 0.08	8.11 ± 0.09	8.02 ± 0.12	8.09 ± 0.05
Subset	7.89 ± 0.20	7.33 ± 0.17	8.00 ± 0.29	7.89 ± 0.20	7.78 ± 0.11
*Russian*					
All bees	8.25 ± 0.08	7.86 ± 0.12	8.37 ± 0.12	7.67 ± 0.11	8.03 ± 0.06
Subset	7.89 ± 0.26	7.44 ± 0.18	7.67 ± 0.17	7.33 ± 0.17	7.58 ± 0.10
*Saskatraz*					
All bees	7.94 ± 0.09	7.98 ± 0.09	7.65 ± 0.07	7.60 ± 0.08	7.80 ± 0.04
Subset	7.67 ± 0.17	7.78 ± 0.15	7.11 ± 0.11	7.00 ± 0.00	7.39 ± 0.08
Avg. treatment					
All bees	8.13 ± 0.04	7.85 ± 0.04	7.79 ± 0.04	7.61 ± 0.04	7.85 ± 0.02
Subset	7.64 ± 0.09	7.44 ± 0.07	7.36 ± 0.09	7.36 ± 0.08	7.45 ± 0.04

**TABLE 5:** Days to emergence ANOVA. ANOVA summary of the generalized linear mixed model fixed effects for the number of days until adult emergence. All viral data were collected from head tissues (N = 180 bees and N = 180 RNA extractions in total). Supplementary Table S1 for model parameter estimates.

Variable	SumSq	MeanSq	NumDF	DenDF	*F*-value	*p*-value
Stock	1.63	0.41	4	10.04	2.05	0.162
Treatment	1.46	0.49	3	150.28	2.46	0.065
Stock*treatment	6.80	0.57	12	147.27	2.86	0.001
Log DWV-A	0.06	0.06	1	156.55	0.29	0.591
Log DWV-B	0.91	0.91	1	151.84	4.57	0.034

### 3.3 Differences in Morphologically Deformed Wing Virus Symptoms

#### 3.3.1 Symptom Presence

**FIGURE 5:**
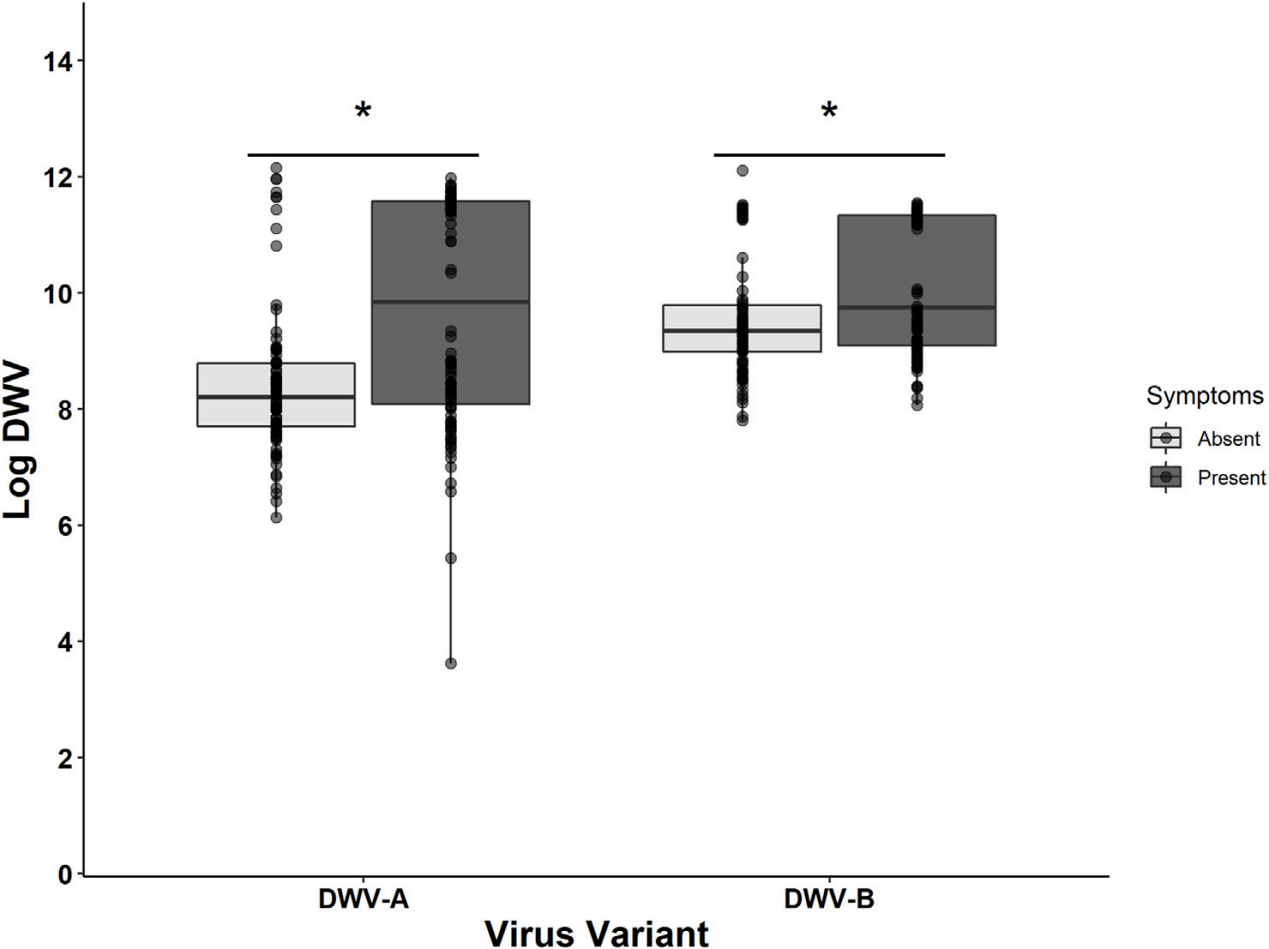
DWV levels given symptom presence. The log-transformed DWV-A and DWV-B levels in head tissues for all sampled bees (N = 180) indicate that bees exhibiting any morphological symptoms had higher levels of both DWV-A and DWV-B. The lighter shade represents asymptomatic bees and the darker shade represents symptomatic bees. Boxplots are in the style of Tukey where the box limits represent the lower 25% quantile and upper 75% quantile, with the line representing the median. * denotes significant differences between asymptomatic and symptomatic values for each DWV variant (Kruskal–Wallis test; *p* < 0.05). For stock-specific comparisons, Supplementary Figure S3.

The treatment group and the DWV variant impacted the probability of emerging bees exhibiting any DWV symptoms (i.e., wing deformities) (Table 6). The treatment group had a significant impact on the presence of symptoms, with DWV injections (associated with increased DWV-A levels) inducing symptoms in a higher number of bees relative to all other treatment groups (compared to control: *z* = 3.962, *p* < 0.001; compared to PBS: *z* = 3.037, *p* = 0.013; compared to mite: *z =* 2.814, *p* = 0.025) (Table 6). Symptomatic bees had higher DWV-A (10^9.71 ± 0.20^) and DWV-B (10^10.14 ± 0.12^) levels than asymptomatic bees (DWV-A: 10^8.45 ± 0.14^; DWV-B: 10^9.57 ± 0.11^) (Figure 5). When DWV levels were compared between symptomatic and asymptomatic bees split out by bee stock, we found that Italian and Pol-Line bees exhibited higher DWV-A levels in symptomatic bees, while only Pol-Line bees exhibited higher DWV-B levels in symptomatic bees (Supplementary Figure S3).

**TABLE 6:** DWV symptom presence logit. Binomial logistic regression fixed effects for the presence of any DWV symptoms (wing deformities). All viral data were collected from head tissues (N = 180 bees and N = 180 RNA extractions in total).

Type	Variable	Estimate	Std. error	*Z*-value	*p*-value
Intercept	Intercept	−5.665	2.043	−2.773	0.006
Stock	Carniolan	0.076	0.567	0.134	0.893
	Pol-Line	1.054	0.578	1.822	0.068
	Russian	0.249	0.562	0.444	0.657
	Saskatraz	0.683	0.561	1.217	0.224
Treatment	PBS	0.869	0.526	1.654	0.098
	Mite	0.945	0.503	1.879	0.060
	DWV	3.064	0.773	3.962	0.000
DWV variant	DWV-A	0.052	0.146	0.357	0.721
	DWV-B	0.383	0.169	2.264	0.024

#### 3.3.2 Symptom Severity

**FIGURE 6:**
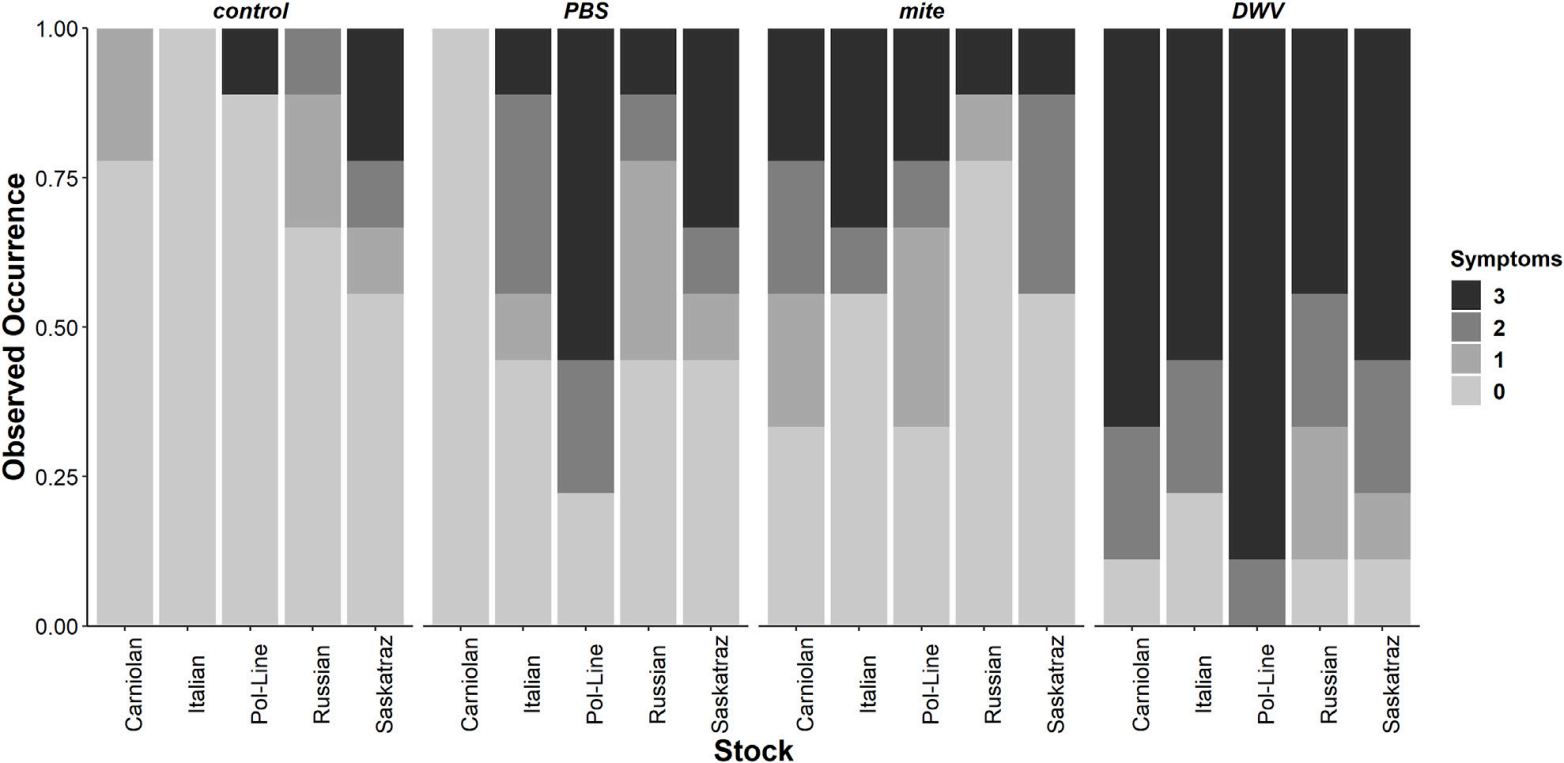
Observed rates of DWV symptom severity. The observed rates of symptom severity for each stock and treatment combination for the subset of bees used for viral analyses (N = 9 bees per stock × treatment combination, N = 180 bees total) indicate significant bee stock × treatment interactions. Contrary to expectations, these interactions are not consistent for all *Varroa* mite–resistant stocks (Pol-Line, Russian, and Saskatraz). Zero represents no symptoms and 3 represents debilitating morphological symptoms based on Supplementary Figure S1. Supplementary Table S4 for mean DWV levels for each bee stock and symptom severity categorization.

When the severity of DWV symptoms (Supplementary Figure S1) was analyzed (Supplementary Table S5), bee stock, treatment group, and stock × treatment group were significant. All bee stocks were significant within the model, indicating potential stock-based differences in DWV tolerance compared to Italian bees–Carniolan (ordered logit parameter estimation, *t =* 15.934, *p* < 0.001), Pol-Line (*t =* 12.980, *p* < 0.001), Russian (*t =* 19.370, *p* < 0.001), and Saskatraz (*t =* 20.278, *p* < 0.001). Stocks varied both in the proportion of observations per symptom severity category (Figure 6) and in their associated DWV-A and -B levels (Supplementary Table S6). Within the ordered logit model (Supplementary Table S5), all treatment groups were significant relative to the control treatment group–mite (*t =* 20.849, *p* < 0.001), PBS (*t =* 20.240, *p* < 0.001), and DWV (*t =* 18.982, *p* < 0.001). DWV injections had the greatest level of symptom severity followed by the PBS and mite treatment groups. No manipulation controls had low-level symptom severity. These same trends mostly held for stock × treatment group interactions but with severity levels differing among stocks within each treatment group (Figure 6). Overall trends from the ordered logit model did a fairly good job predicting symptom severity per treatment and stock; but interpreting precise model results needs to be carried out with care and in conjunction with the following multivariate analyses for a more complete picture as no symptoms were observed for Italian bees in control treatment and Carniolan bees in the PBS treatment group, skewing the ordered logit model values.

#### 3.3.3 Multivariate Analyses of Symptom Outcomes

**FIGURE 7:**
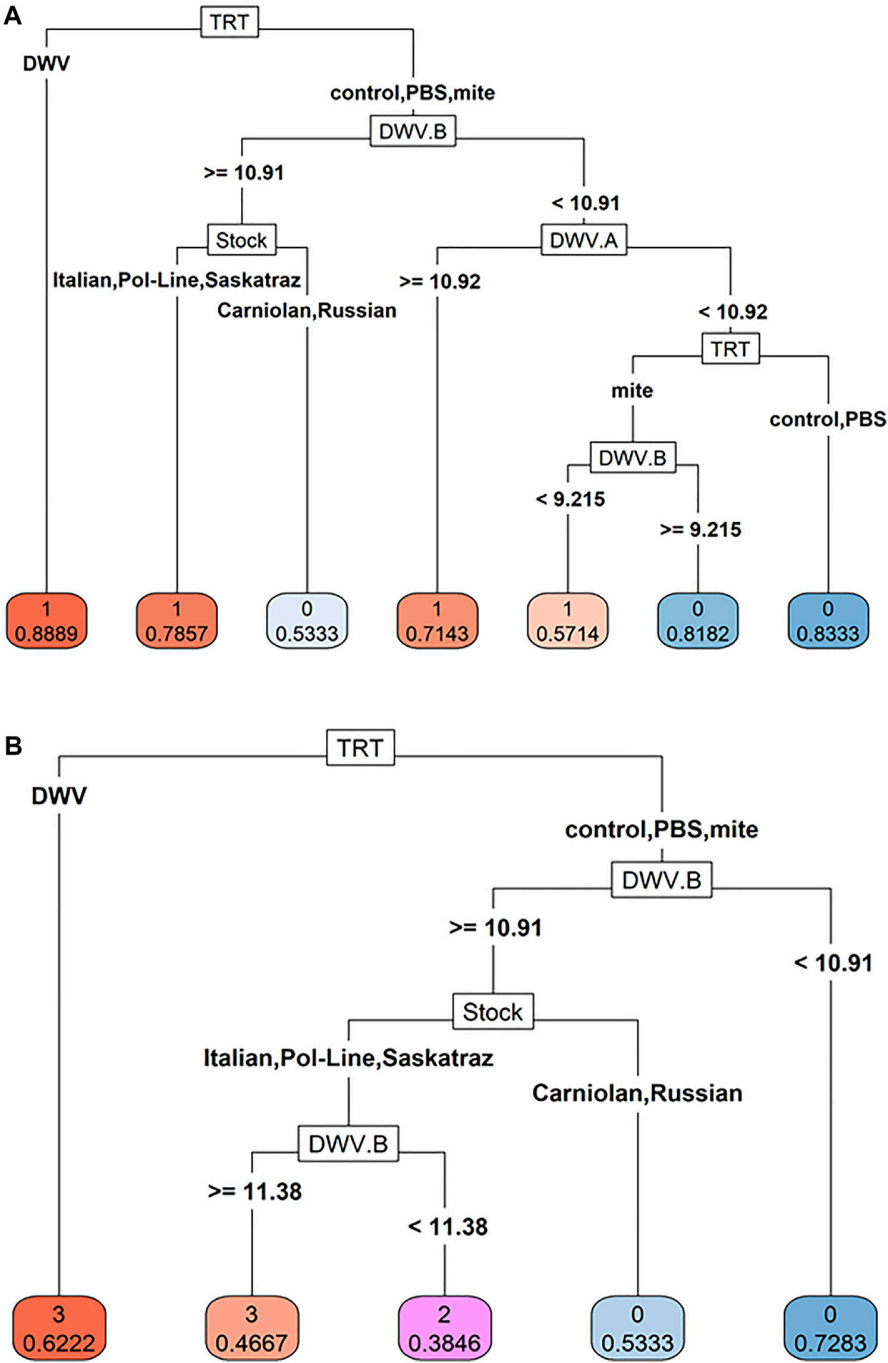
Classification trees of DWV symptoms. Classification trees to analyze **(A)** DWV symptom presence (presence = 1, absence = 0) and **(B)** DWV symptom severity (ordinal scale 0–3, 0 represents no symptoms, and 3 represents debilitating symptoms) were created using bee stock, treatment, log-transformed DWV-A (DWV.A) and DWV-B (DWV.B) levels as predictor variables through recursive partitioning and with a complexity parameter of >0.01 after pruning. The predicted probabilities per decision node are shown beneath each variable categorization (i.e., 0 = asymptomatic, 1 = symptomatic in A; or the scale of 0–3 where 0 represents no symptoms and 3 represents debilitating morphological symptoms based on Supplementary Figure S1). Virus data were based on head tissues (N = 180 bees).

To better understand the relative importance of bee stock, treatment group, and DWV levels for DWV symptoms, we used classification trees to analyze the probability of symptom presence and severity. We found that DWV levels of both variants in combination with bee stock and the treatment group played a critical role in predicting the probability of symptom presence (Figure 7A). In the first decision node of the symptom presence (1)/absence (0) tree, bees with DWV injection had the highest probability of symptoms (0.89). The second decision node split probabilities using DWV-B levels (cut off of 10^10.91^), where bees whose heads contained levels above this cutoff were more likely to exhibit symptoms. The next two nodes indicated equal importance of DWV-A levels (cut off of 10^10.92^) and bee stock. This node grouped Italian, Pol-Line, and Saskatraz bees together as being more likely to exhibit symptoms (>78% of bees likely to show symptoms) and Carniolan and Russian bees together as less likely to exhibit symptoms (>53% of bees likely to not exhibit morphological symptoms).

The classification tree for symptom severity (Figure 7B) was more simplified than that for symptom presence. Like the symptom presence tree, the first decision node in the severity tree was the DWV injection treatment group and the second was the level of DWV-B (with the same cut off of 10^10.91^). The third node was solely based on honey bee stock, again grouping Italian, Pol-Line, and Saskatraz bees together, indicating that these stocks have an increased probability of having more severe symptoms than Carniolan and Russian stocks. DWV-A levels did not explicitly play a part in the symptom severity tree, but DWV-A levels may have been partially accounted for through the DWV injection treatment as DWV-A levels increased in this treatment group whereas DWV-B levels did not (Table 1; Figure 2).

**FIGURE 8:**
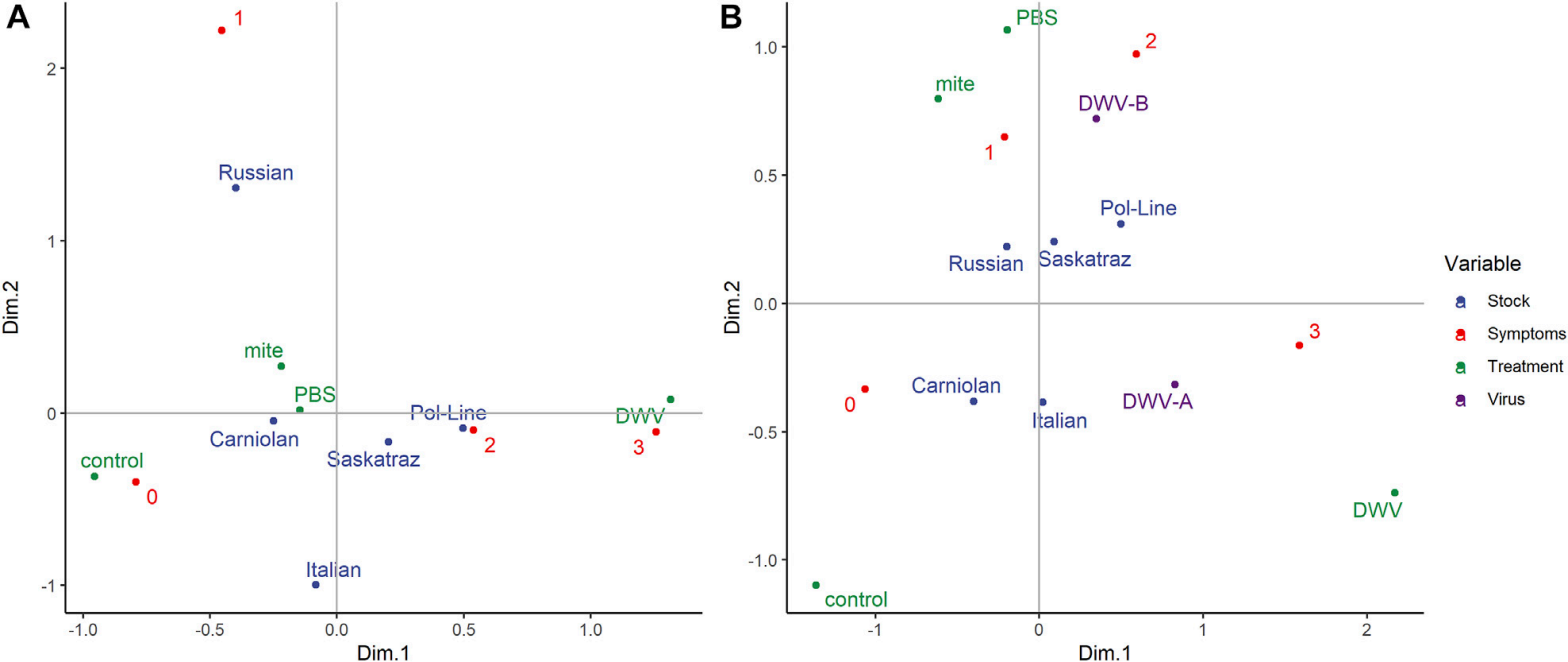
Multivariate visualization of bee stock, treatments, and symptom severity. **(A)** Multiple correspondence analysis to evaluate the interactions among the following categorical variables: bee stocks, treatments, and DWV symptom severity (scale 0–3, 0 represents no symptoms, and 3 represents debilitating morphological symptoms based on Supplementary Figure S1). **(B)** Factor analysis of mixed data to evaluate the interactions among five bee stocks, four treatment groups, and DWV symptom severity with the addition of log-transformed levels (virus) of both DWV variants (A and B). Both figures represent the correlations between variables, where variables with similar profiles group together. Sample sizes were based on bees with DWV quantification from head tissues (N = 180 bees).

Using multiple correspondence analysis (MCA) to further validate the relationships among stock, treatment group, and symptom severity (Figure 8A), we found that treatment groups fell along a gradient of symptom severity on dimension 1 (Dim.1: 0.685). DWV injections were represented at the far positive end of Dim.1, and no manipulation controls at the far negative end with mite and PBS injection treatment groups being intermediate along this dimension. Symptom severity (Dim.1: 0.790 and Dim.2: 0.602) loaded onto both dimensions/axes while bee stock loaded primarily on Dim.2 (Dim.1: 0.105, Dim.2: 0.548). Notably, Carniolan, Italian, and Russian stocks loaded on the negative end of Dim.1, while Pol-Line and Saskatraz bees load onto the positive end of Dim.1. Furthermore, the Russian bees were the only stock to load positively onto Dim.2. Overall, Dim.1 explained 15.8% of the variation in the data, while Dim.2 accounted for 12.1%. Dimensions 3–5 also saw significant loadings of the bee stock and treatment group variables.

When we used factor analysis of mixed data (FAMD) (Figure 8B) to add DWV-A and DWV-B levels to the multivariate analysis, Dim.1 accounted for 18.5% of the variation in the data, while Dim.2 accounted for 12.1%, with the variables of DWV-A (Dim.1: 30.88 and Dim.2: 6.79) and DWV-B (Dim.1: 5.43 and Dim.2: 35.54) being the primary contributors. Dimensions 3 and 4 had significant loadings of the bee stock (Dim.3: 48.66 and Dim.4: 61.53) and symptom severity (Dim.3: 48.82 and Dim.4: 32.73). In FAMD, the grouping by stock shifted such that Italian bees joined the Pol-Line and Saskatraz grouping with more severe symptoms (positive along Dim.1), while Carniolan and Russian bees still grouped together with less severe symptoms (negative along Dim.1). This grouping of stocks was consistent with the classification tree results for both symptom presence and severity. However, the stocks and treatment groups can also be grouped by DWV levels, where Pol-Line, Russian, and Saskatraz bees (the three mite-resistant stocks within the study) and mite and PBS treatment groups were grouped with DWV-B on the positive end of Dim.2, while Carniolan and Italian bees loaded on the negative end of Dim.2 with DWV-A.

## 4 Discussion

The goal of this study was to determine if *Varroa* mite–resistant honey bee stocks (Pol-Line, Russian, and Saskatraz) exhibited differential resistance or tolerance to DWV compared to mite-susceptible stocks (Carniolan and Italian). We exposed white-eyed pupae to mites, injected them with a sublethal dose (10^5^) of DWV obtained from symptomatic adults, injected them with PBS as sham injection, or did not manipulate them at all as a control. After adult emergence, we evaluated dissemination of DWV variants A and B throughout four tissue types and adult emergence time and symptom severity. The DWV variants differed in their dissemination patterns and associations with treatment groups and, potentially, in their contributions to symptom severity. While bee stocks did not differ in overall virus RNA copy levels or dissemination patterns associated with either DWV variant (not indicative of resistance), bee stock interacted with the treatment group and the DWV level to impact symptom severity (indicative of tolerance). However, these stock interactions were not consistently correlated with known mite resistance.

The levels of both DWV variants differed with the treatment group but not in relation to bee stock, indicating that, in this study, stocks were not resistant to DWV unlike the complementary study on injected adults where some stocks appeared to exhibit DWV resistance (Penn et al., 2021). Sublethal pupal injections of DWV (inoculum obtained from symptomatic adults screened using general DWV primers) induced elevated levels of DWV-A but not DWV-B, which is also dissimilar to responses in DWV-injected adults from different cohorts collected within the same timeframe from the same colonies using an aliquot from the same inoculum (both variants were elevated with DWV injections) (Penn et al., 2021). Conversely, PBS injection and mite exposure pupal treatment groups induced higher levels of DWV-B but not DWV-A (Figure 2). The elevated levels of DWV-B in the PBS and mite treatment groups are also similar to prior responses seen in adult bees but not necessarily in pupae (Wu et al., 2017; Dubois et al., 2020; Norton et al., 2020; Penn et al., 2021; Ray et al., 2021). The differential responses to viral infection between pupae and adults have been previously documented both in infection levels and in the ability of dsRNA treatments to mitigate infections (Möckel et al., 2011; Desai et al., 2012; Thaduri et al., 2019). Such ontogenetic responses to viral infections are likely related to differences in immune gene regulation among life stages, with the introduction of infection or stress at certain points such as the pupal stage being more critical due to lower levels of immune response (Wilson-Rich et al., 2008; Laughton et al., 2011; Koleoglu et al., 2017). These results highlight the importance of including life stage as a factor when investigating or selecting for viral resistance.

Despite having no symptoms and no prior exposure to *Varroa* mites, the untreated control group bees exhibited some DWV infection, possibly from vertical transmission or horizontal transmission in larval food as mites were not present within the pupal cells (Yue et al., 2007; Möckel et al., 2011). Therefore, the increased levels of DWV in the PBS group compared to those of the control group are most likely due to injection trauma–inducing viral replication (Gusachenko et al., 2020; Penn et al., 2021). The *Varroa* mites used in this study had greater DWV-B levels (10^4.82 ± 0.20^) than DWV-A (10^2.42 ± 0.34^), with similar DWV levels observed in previous studies (Gisder et al., 2009; Wu et al., 2017; Tentcheva et al., 2018). However, the observed mite DWV levels were not correlated with the associated bee levels for either DWV variant (unlike Wu et al., 2017). Mites may lose their DWV infection quite rapidly, particularly if mites feed on bees with low virus levels (Posada-Florez et al., 2019); and different mite loads do not necessarily increase bee DWV levels but may be associated with certain DWV variants (Ryabov et al., 2014). Given these data and that the mites were fed on pupae exchanged daily for 24–48 h prior to experimental use, the similar responses to the mite and PBS injection treatment groups in this study are not unprecedented.

Patterns of DWV dissemination to different tissue types following pupal exposure varied with the virus variant and the treatment group but not bee stock, reflecting complementary data on injected adult bees from the same colonies (Penn et al., 2021). For dissemination, DWV infection of both variants was readily evident in bee heads, consistent with work showing high DWV infections within the head particularly in overt infections (Shah et al., 2009; Zioni et al., 2011; Penn et al., 2021). The levels of DWV in other tissue types were variant-dependent. Unless directly injected with DWV, abdomens had lower levels of DWV-A than other tissue types (Figure 3) but had levels of DWV-B matching that of other tissues in the mite and PBS treatment groups (Penn et al., 2021). Treatment group differences in dissemination may indicate that replication of latent viral infections may not only be induced by non-viral stressors but also depend on virus variant and bee immune responses (Anderson and Gibbs, 1988; Yang and Cox-Foster, 2005; Boncristiani et al., 2013; Kuster et al., 2014; Khongphinitbunjong et al., 2015; Annoscia et al., 2019; Remnant et al., 2019; Tehel et al., 2019; Mookhploy et al., 2021). For instance, phenol oxidase and antimicrobial peptides have been shown to be upregulated when bees were injected with DWV but not PBS compared to controls, whereas hemocyte counts increased following both DWV and PBS injections (Ryabov et al., 2016; Millanta et al., 2019).

Both DWV variants contributed to symptom presence – DWV-B levels and DWV injection (associated with elevated DWV-A levels) were significant in the model (Table 5) and the symptom presence/absence classification tree (Figure 7A), similar to prior work (Tehel et al., 2019; Dubois et al., 2020). Symptom severity was linked to all non-control treatment groups in the model (DWV-A associated with DWV injection and DWV-B associated with PBS injection and mite exposure) (Supplementary Table S5). Though previous data on how DWV levels impact symptom severity have had mixed results (Möckel et al., 2011; Desai et al., 2012; Tehel et al., 2019; Dubois et al., 2020; Yañez et al., 2020; Mookhploy et al., 2021), we observed that the DWV treatment group (elevated DWV-A) and higher levels of DWV-B were associated with the most severe symptoms (Figure 7). The two variants may also differ in which symptom severity they cluster with (Figure 8B) where DWV-A (and the DWV treatment group) clusters with the severity of 3 (most severe), whereas DWV-B clusters closer severities 1 and 2. Variant-related differences in mortality (not measured here)—where DWV-A-infected bees had higher mortality than DWV-B infected bees—have also been documented in Australian, DWV-naïve bee populations (Norton et al., 2020). Using the classification tree data (Figure 7), we found that DWV levels of 10^10.91^ appeared to be the initial cutoff point for symptom presence and severity. However, these values varied more with the variant in the symptom logit model; symptomatic bees had an average of 10^9.71 ± 0.20^ for DWV-A and 10^10.14 ± 0.12^ for DWV-B compared to asymptotic bees with an average of 10^8.45 ± 0.14^ DWV-A and 10^9.57 ± 0.11^ DWV-B. The two DWV variants were also significantly positively correlated with each other as seen in similar studies of both injected pupae and adults (Dubois et al., 2020; Penn et al., 2021), potentially reflecting the presence of variant recombinants that will need to be addressed in future work (Gisder et al., 2018; Jamnikar-Ciglenecki et al., 2019; Martin and Brettell, 2019).

Bee stock differences could exacerbate symptoms and infection levels after viral exposure as immune responses and rates of both pupal and adult exposure have been shown to shift with host genetics (Santillán-Galicia et al., 2010; Laughton et al., 2011; Möckel et al., 2011; Chang et al., 2021; Hinshaw et al., 2021; Posada-Florez et al., 2021; Weaver et al., 2021). For instance, hygienic bees may have higher exposure rates to DWV than non-hygienic bees since hygienic bees exhibit increased rates of cannibalism on mite-infested pupae (Posada-Florez et al., 2021). When we considered adult emergence time and DWV symptom presence and severity, bee stocks appeared to differ in their tolerance to DWV; though these results were not necessarily consistent with stocks bred for mite resistance (Khongphinitbunjong et al., 2016; Locke et al., 2021). In the multivariate and classification tree analyses (Figures 7, 8), the Russian stock most consistently grouped with Carniolan as being DWV-tolerant (high level of the virus with fewer symptoms and/or lower severity), while Pol-Line grouped with Italian and Saskatraz as more DWV susceptible, particularly when considering symptom severity (Dim.1 axis on Figures 8A,B). However, when focusing on DWV-B levels (Figure 8B), the stock groupings were consistent with mite resistance (positive on Dim.2 axis). Russian, Pol-Line, and Saskatraz grouped near the higher DWV-B loading and the mite and PBS treatment groups on the positive side of Dim.2 in the FAMD (Figure 8B). As prior work has shown that mites are more likely to be infected with DWV-B compared to DWV-A (Gisder and Genersch, 2021), we might expect that mite-resistant stocks would group together when based on variant B.

Differentiation of mite-resistant stocks from each other is not surprising based on some limited prior research (Khongphinitbunjong et al., 2016; Penn et al., 2021) and given their different selection regimens (Rinderer et al., 2010; Robertson et al., 2014; Danka et al., 2016; Saelao et al., 2020). The separation of Pol-Line from Russian and Carniolan bees is similar to the grouping by genetic sequencing conducted by Saelao et al. (2020), where Pol-Line splits into a separate group from Carniolan, Italian, and Russian stocks (Saelao et al., 2020). Although Saelao et al. (2020) did not include Saskatraz bees in their analysis and our Italian bees did not segregate similarly to theirs in our analyses, it appears that the Pol-Line separation from Carniolan and Russian bees may be due, in part, to differential susceptibility to DWV in addition to genetic clustering for other traits (e.g., the strong selection for *Varroa* sensitive hygienic behavior). When we compared results from this study and the complementary study on injected adult bees from the same colonies (Penn et al., 2021), injected Pol-Line adults exhibited the greatest resistance to DWV-A compared to the other bee stocks but had the lowest tolerance following DWV injections of pupae in this study (Figures 6–8). Russian bees appeared tolerant to DWV overall as injected adults had the highest levels of DWV-A in the other study and the greatest tolerance in this study. These data indicate that there might be tradeoffs in breeding for mite resistance, DWV resistance, and symptom expression or tolerance. Regardless of the particular mechanisms underpinning these differences, for example, physiological responses to DWV or prevention of *Varroa* parasitism and DWV exposure (Navajas et al., 2008; Khongphinitbunjong et al., 2015; Posada-Florez et al., 2021), stock-related virus resistance or tolerance may help account for differences in colony survival in the field (Locke et al., 2014; Thaduri et al., 2019).

## 5 Conclusion

These data suggest that the genetic stock of the honey bee host is important for viral tolerance when pupae are exposed to DWV and other stressors but is not always perfectly aligned with breeding for mite resistance. Furthermore, we found that DWV variants differ in their dissemination in newly emerged adults and contribute significantly to DWV symptom presence though differentially to symptom severity. More severe symptoms were associated with DWV injections that elevated DWV-A levels, while less severe symptoms were associated with DWV-B, injection injury, and mite feeding. Given prior work on the same colonies showing that certain stocks of injected adults bees are more resistant to DWV-A infection over time, further study into the timing of infection and stock-mediated immune responses is vital to unraveling how genetic stocks interact with DWV. Comparison of these studies also indicates that more study of isolated viral variants rather than the naturally occurring combination of variants from overtly symptomatic adults is needed to parse out the full physiological impacts of each variant. More broadly, this research continues to highlight the importance of considering the combination of host genotype and pathogen genotype in epidemiological studies.

## Data Availability

The raw data supporting the conclusion of this article will be made available by the authors, without undue reservation.
