# Supplementary material for: Honey Bee Genetic Stock Determines Deformed Wing Virus Symptom Severity but not Viral Load or Dissemination Following Pupal Exposure
Source: Front Genet. 2022 Jun 3;13:909392. doi: 10.3389/fgene.2022.909392 (PMC9204523; doi:10.3389/fgene.2022.909392)
Supplement: Supplementary file 1 [file DataSheet1.docx]

Supplementary Material

**Supplementary Figure 1. DWV Symptom classification.** Morphological classifications are used to determine DWV symptom severity of emerging adult bees.

**
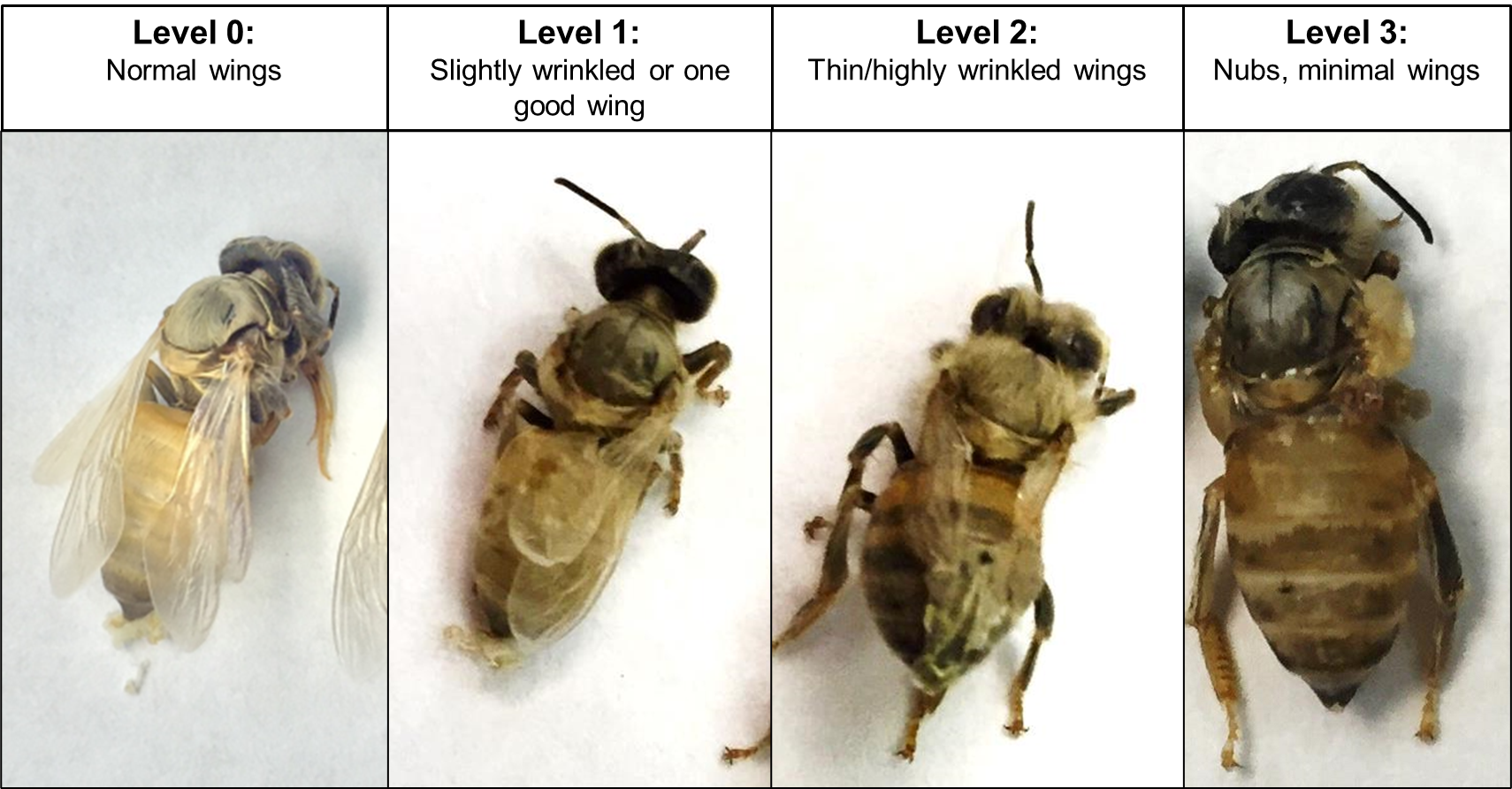
**

**Supplementary Figure 2. Experiment differences in DWV.** Log-transformed **(A)** DWV-A and **(B)** DWV-B levels found in all tissues of bees from five stocks (colors) compared between experiments (N = 135 bees per experiment). The pupae experiment represents data described in this manuscript (darker shades at right) while the adult data represent day 7 samples of injected newly emerged adults (lighter shades at left) from [Penn et al. 2021](https://doi.org/10.3389/finsc.2021.756690). Boxplots are in the style of Tukey where the box limits represent the lower 25% quantile and upper 75% quantile with the line representing the median. *P*-values are from Kenward-Rogers post-hoc contrasts from the associated model results (Table 3) of adult versus pupae experiments within each treatment but pooling bee stocks. Generally, newly emerged adults treated as pupae had higher DWV-A levels than the day 7 adults treated as newly emerged adults. Conversely, the injected adults had higher levels of DWV-B than the injected pupae; but this was only significant in the DWV treatment.


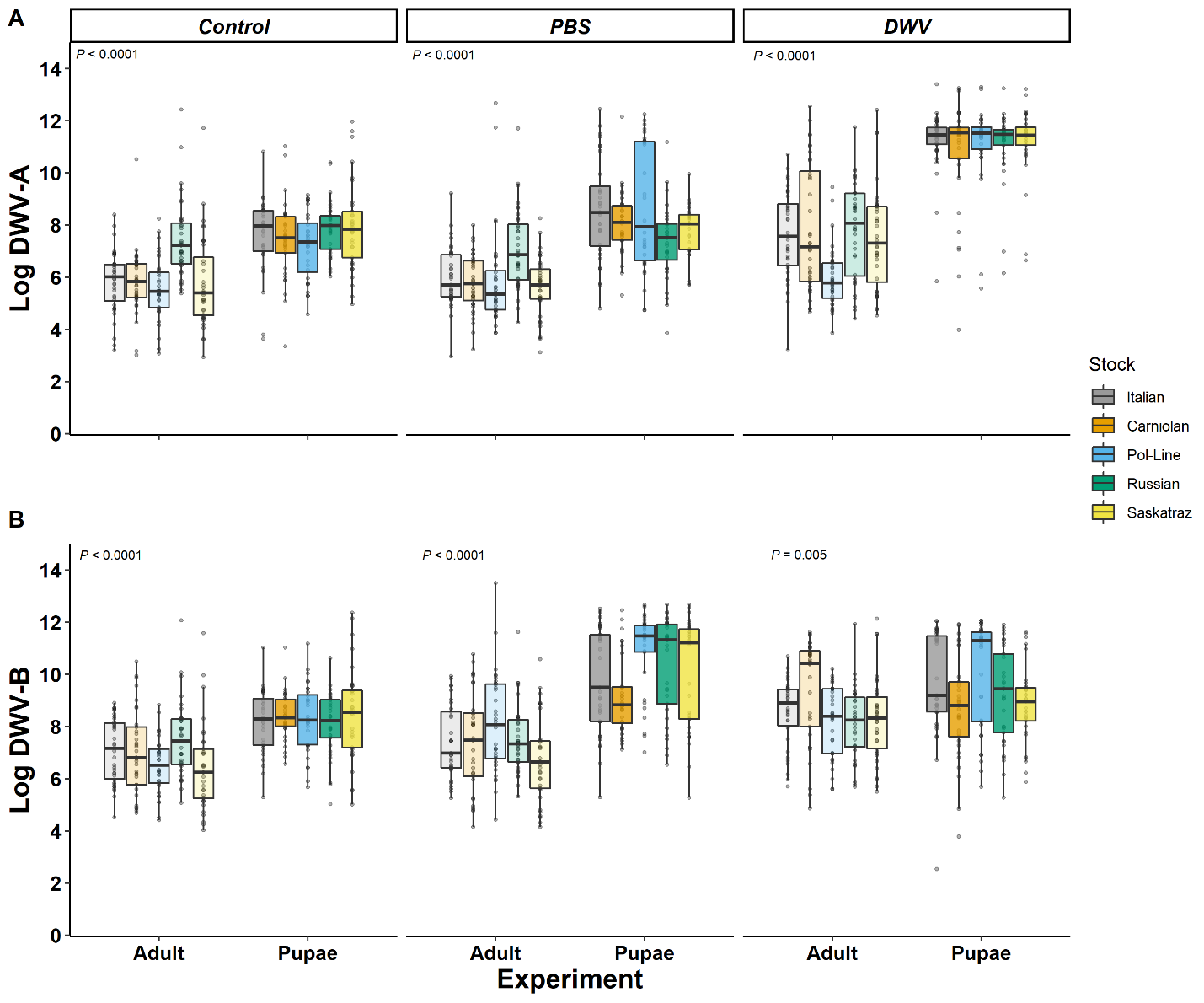


**Supplementary Figure 3. DWV levels higher with symptom presence.** The log-transformed (A) DWV-A and (B) DWV-B levels in head tissue for each bee stock (color) where lighter shades (left) represent asymptomatic bees and darker shades (right) represent symptomatic bees. Boxplots are in the style of Tukey where the box limits represent the lower 25% quantile and upper 75% quantile with the line representing the median. Points indicate samples from individual bees with point shapes indicating the colony replicate (ColonyRep) within the associated bee stock (N = 36 bees per stock). The number of individuals per stock and category can be found in S3 Table. * Denotes significant differences between asymptomatic and symptomatic values for each stock (Kruskal-Wallis test; *P* < 0.05). Only Pol-Line bees had higher levels of DWV-A and B in morphologically symptomatic bees compared to asymptotic bees, while Italian bees only exhibited higher DWV-A but not B levels in symptomatic bees. Dotted lines indicate average levels (across stocks) for asymptomatic bees while dashed lines indicate the average for symptomatic bees.


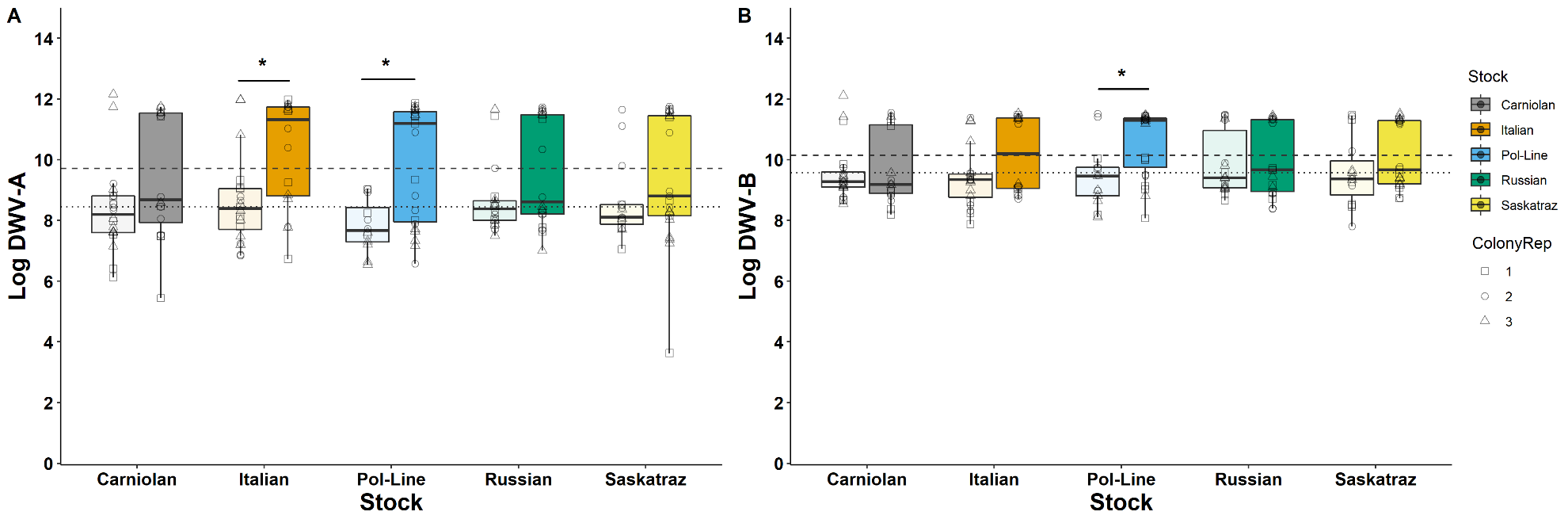


**Supplementary Table 1.** **PCR Primers.** Primers used for virus detection and sample quality.

| Primer Target | Forward Sequence | Reverse Sequence | Reference |
| --- | --- | --- | --- |
| AKI (ABPV, KBV, and IAPV combination) | ACCGACAAAGGGTATGATGC | CTTGAGTTTGCGGTGTTCCT | Roy et al. J Apic Sci DOI:10.2478/v10289-012-0014-x |
| BQCV | TTTAGAGCGAATTCGGAAACA | GGCGTACCGATAAAGATGGA | Boncristiani et al. 2012, J Ins Phys  [DOI:10.1016/j.jinsphys.2011.12.011](https://doi-org.nal.idm.oclc.org/10.1016/j.jinsphys.2011.12.011) |
| CBPV | CGCAAGTACGCCTTGATAAAGAAC | ACTACTAGAAACTCGTCGCTTCG | Blanchard et al. 2007, J Virol Meth  [DOI:10.1016/j.jviromet.2006.11.021](https://doi-org.nal.idm.oclc.org/10.1016/j.jviromet.2006.11.021) |
| DWV, non-specific | GGACCATCCTTCCAGTCTACGAT | CTGTAGGTTGTGCTCCTGATGAAGA | Ryabov et al. 2014, Plos Pathogens [DOI:10.1371/journal.ppat.1004230.s008](https://doi.org/10.1371/journal.ppat.1004230.s008) |
| DWV-A | GAGATTGAAGCGCATGAACA | TGAATTCAGTGTCGCCCATA | Boncristiani et al. 2012, J Ins Phys  [DOI:10.1016/j.jinsphys.2011.12.011](https://doi-org.nal.idm.oclc.org/10.1016/j.jinsphys.2011.12.011) |
| DWV-B | CTGTAGTTAAGCGGTTATTAGAA | GGTGCTTCTGGAACAGCGGAA | Ryabov et al. 2014, Plos Pathogens [DOI:10.1371/journal.ppat.1004230.s008](https://doi.org/10.1371/journal.ppat.1004230.s008) |
| LSV | CGTGCGGACCTCATTTCTTCATGT | CTGCGAAGCACTAAAGCGTT | Daughenbaugh et al. 2015, Viruses  [DOI:10.3390/v7062772](https://doi.org/10.3390/v7062772) |
| β-actin | AGGAATGGAAGCTTGCGGTA | AATTTTCATGGTGGATGGTGC | Ryabov et al. 2014, Plos Pathogens [DOI:10.1371/journal.ppat.1004230.s008](https://doi.org/10.1371/journal.ppat.1004230.s008) |

**Supplementary Table 2.** **Models.** An overview of the model, R package information, and equation for each section.

| **Model** | **Package Information** | **Equation** |
| --- | --- | --- |
| Dissemination | Lme4, lmer, Gaussian | DWV.A/B ~ Stock + TRT + Dissection + DWV.B/A + Dissection*TRT  + (1\|BeeID) + (1 \| Colony) |
| Experiment Comparison | Lme4, lmer, Gaussian | DWV.A ~ Stock + TRT + Experiment + Dissection  + Stock*TRT + Experiment*Stock + Stock*Dissection  + Experiment*TRT + Stock*TRT*Experiment  + TRT*Dissection + Experiment*Dissection  + TRT*Experiment*Dissection  + (1 \| Colony) + (1\|BeeID) |
| Emergence Time | Lme4, lmer, Gaussian | DaysToEmerge ~ Stock + TRT + Stock*TRT + DWV.A + DWV.B  + (1 \| Colony) |
| Symptom Presence | Lme4, lmer, Binomial | Symptoms_PA ~ Stock + TRT + DWV.A + DWV.B + (1 \| Colony) |
| Symptom Severity | MASS, polr, Logit | Symptoms_scale ~ Stock + TRT + Stock * TRT + DWV.A + DWV.B |

**Supplementary Table 3. DWV dissemination GLMMs.** Generalized linear mixed model fixed effects for the log-transformed DWV-A and DWV-B titers disseminated throughout the adult tissues (N = 180 bees total and N = 720 RNA extractions in total).

|  | | Log DWV-A | | | | Log DWV-B | | | |
| --- | --- | --- | --- | --- | --- | --- | --- | --- | --- |
| Type | Variable | Sum Sq | Mean Sq | *t*-value | *P*-value | Estimate | Std. Error | *t*-value | *P*-value |
| Intercept | Intercept | 7.702 | 0.516 | 14.923 | 0.000 | 7.910 | 0.502 | 15.746 | 0.000 |
| Stock | Carniolan | -0.452 | 0.475 | -0.951 | 0.341 | -0.098 | 0.426 | -0.231 | 0.818 |
|  | Pol-Line | -0.101 | 0.476 | -0.213 | 0.831 | 0.582 | 0.426 | 1.367 | 0.172 |
|  | Russian | -0.219 | 0.475 | -0.461 | 0.645 | 0.236 | 0.425 | 0.554 | 0.579 |
|  | Saskatraz | -0.172 | 0.475 | -0.363 | 0.717 | 0.335 | 0.425 | 0.787 | 0.432 |
| Treatment (TRT) | PBS | 0.310 | 0.307 | 1.011 | 0.312 | 1.147 | 0.370 | 3.105 | 0.002 |
|  | Mite | -0.117 | 0.304 | -0.385 | 0.700 | 0.856 | 0.368 | 2.325 | 0.020 |
|  | DWV | 3.199 | 0.304 | 10.533 | 0.000 | 0.364 | 0.390 | 0.935 | 0.350 |
| Tissue (TIS) | Abdomen (A) | -1.312 | 0.251 | -5.223 | 0.000 | -1.456 | 0.227 | -6.421 | 0.000 |
|  | Hyp. Gland (G) | -0.614 | 0.281 | -2.184 | 0.029 | -1.061 | 0.253 | -4.185 | 0.000 |
|  | Leg (L) | -0.186 | 0.242 | -0.769 | 0.442 | -0.858 | 0.217 | -3.961 | 0.000 |
| TRT × TIS | PBS - A | 0.084 | 0.356 | 0.235 | 0.814 | 1.273 | 0.319 | 3.993 | 0.000 |
|  | Mite - A | 0.117 | 0.345 | 0.338 | 0.736 | 0.986 | 0.310 | 3.184 | 0.001 |
|  | DWV - A | 1.314 | 0.344 | 3.813 | 0.000 | -0.088 | 0.315 | -0.279 | 0.780 |
|  | PBS - G | -0.450 | 0.392 | -1.149 | 0.251 | 0.309 | 0.356 | 0.868 | 0.386 |
|  | Mite - G | 0.335 | 0.392 | 0.856 | 0.392 | 0.931 | 0.355 | 2.626 | 0.009 |
|  | DWV - G | -1.514 | 0.390 | -3.880 | 0.000 | 0.498 | 0.359 | 1.388 | 0.165 |
|  | PBS - L | 0.120 | 0.341 | 0.352 | 0.725 | 0.522 | 0.307 | 1.698 | 0.089 |
|  | Mite – L | 0.203 | 0.340 | 0.597 | 0.550 | 0.378 | 0.306 | 1.235 | 0.217 |
|  | DWV - L | 0.423 | 0.340 | 1.244 | 0.213 | 0.437 | 0.307 | 1.425 | 0.154 |
| DWV Strain | DWV-A |  |  |  |  | 0.128 | 0.039 | 3.277 | 0.001 |
|  | DWV-B | 0.075 | 0.038 | 2.007 | 0.045 |  |  |  |  |

**Supplementary Table 4. Days to emergence GLMM.** General linear mixed model fixed effects for the number of days until adult emergence. All viral data were collected from head tissues (N = 180 bees and N = 180 RNA extractions in total.

| Type | Variable | Estimate | Std. Error | *t*-value | *P*-value |
| --- | --- | --- | --- | --- | --- |
| Intercept | Intercept | 8.073 | 0.466 | 17.310 | 0.000 |
| Stock | Carniolan (C) | -0.314 | 0.304 | -1.033 | 0.302 |
|  | Pol-Line (P) | 0.347 | 0.304 | 1.141 | 0.254 |
|  | Russian (R) | 0.330 | 0.303 | 1.088 | 0.276 |
|  | Saskatraz (S) | 0.138 | 0.304 | 0.453 | 0.650 |
| Treatment (TRT) | PBS | -0.155 | 0.214 | -0.726 | 0.468 |
|  | Mite | -0.497 | 0.212 | -2.350 | 0.019 |
|  | DWV | -0.324 | 0.239 | -1.354 | 0.176 |
| TRT × Stock | PBS – C | 0.288 | 0.298 | 0.966 | 0.334 |
|  | Mite – C | 0.342 | 0.297 | 1.152 | 0.249 |
|  | DWV – C | 0.399 | 0.297 | 1.341 | 0.180 |
|  | PBS – P | -0.233 | 0.303 | -0.768 | 0.442 |
|  | Mite – P | 0.664 | 0.297 | 2.236 | 0.025 |
|  | DWV – P | 0.364 | 0.297 | 1.224 | 0.221 |
|  | PBS – R | -0.161 | 0.299 | -0.539 | 0.590 |
|  | Mite – R | 0.342 | 0.297 | 1.151 | 0.250 |
|  | DWV – R | -0.215 | 0.297 | -0.724 | 0.469 |
|  | PBS – S | 0.332 | 0.299 | 1.112 | 0.266 |
|  | Mite – S | 0.014 | 0.299 | 0.048 | 0.961 |
|  | DWV – S | -0.385 | 0.298 | -1.290 | 0.197 |
| DWV Strain | DWV-A | 0.018 | 0.034 | 0.538 | 0.591 |
|  | DWV-B | -0.074 | 0.035 | -2.138 | 0.033 |

**Supplementary Table 5. Symptom severity ordered logit.** Ordered logit model fixed effects for DWV symptom severity (scale of 0-3). Italian stock and control treatment are used as intercept values. Titer levels are indicative of head tissues only (N = 180 bees and N = 180 RNA extractions in total). Interpreting model results needs to be done with care as few symptoms were observed for Italian bees in the control treatment and Carniolan bees in the PBS treatment (see Fig. 5), skewing these values.

| Type | Variable | Estimate | Std. Error | *t*-value | *p*-value |
| --- | --- | --- | --- | --- | --- |
| Stock | Carniolan (C) | 12.649 | 0.794 | 15.934 | 0.000 |
|  | Pol-Line (P) | 12.096 | 0.932 | 12.980 | 0.000 |
|  | Russian (R) | 13.241 | 0.684 | 19.370 | 0.000 |
|  | Saskatraz (S) | 13.881 | 0.685 | 20.278 | 0.000 |
| Treatment (TRT) | PBS | 13.936 | 0.689 | 20.240 | 0.000 |
|  | Mite | 14.091 | 0.676 | 20.849 | 0.000 |
|  | DWV | 15.477 | 0.815 | 18.982 | 0.000 |
| TRT × Stock | PBS – C | -27.905 | 0.000 | -6579508 | 0.000 |
|  | Mite – C | -12.261 | 1.085 | -11.300 | 0.000 |
|  | DWV – C | -11.982 | 1.132 | -10.587 | 0.000 |
|  | PBS – P | -10.262 | 1.240 | -8.277 | 0.000 |
|  | Mite – P | -11.998 | 1.180 | -10.167 | 0.000 |
|  | DWV – P | -10.282 | 1.443 | -7.127 | 0.000 |
|  | PBS – R | -13.603 | 1.005 | -13.542 | 0.000 |
|  | Mite – R | -14.761 | 1.125 | -13.122 | 0.000 |
|  | DWV – R | -13.598 | 0.995 | -13.669 | 0.000 |
|  | PBS – S | -13.588 | 1.029 | -13.205 | 0.000 |
|  | Mite – S | -14.265 | 1.039 | -13.734 | 0.000 |
|  | DWV – S | -13.753 | 1.023 | -13.448 | 0.000 |
| DWV Strain | DWV-A | 0.064 | 0.139 | 0.463 | 0.643 |
|  | DWV-B | 0.243 | 0.157 | 1.544 | 0.123 |
| Symptom Severity  Intercepts | 0 - 1 | 16.795 | 1.884 | 8.916 | 0.000 |
|  | 1 - 2 | 17.485 | 1.896 | 9.221 | 0.000 |
|  | 2 -3 | 18.445 | 1.908 | 9.665 | 0.000 |

**Supplementary Table 6. DWV levels given symptom severity.** Mean log-transformed DWV-A and DWV-B levels (± standard error of the mean) for all symptom severity categories where 0 represents no symptoms and 3 represents debilitating symptoms. Data are broken down for each bee stock within each symptom category with the number of individuals (N) with the associated symptom severity listed in parentheses for each stock. All viral data were collected from head tissues.

| Symptom Severity | Bee Stock (N) | Log DWV-A | Log DWV-B |
| --- | --- | --- | --- |
| 0 | Carniolan (20) | 8.374 ± 0.33 | 9.557 ± 0.21 |
|  | Italian (20) | 8.625 ± 0.33 | 9.418 ± 0.23 |
|  | Pol-Line (13) | 7.819 ± 0.25 | 9.470 ± 0.29 |
|  | Russian (18) | 8.652 ± 0.27 | 9.843 ± 0.25 |
|  | Saskatraz (15) | 8.585 ± 0.33 | 9.570 ± 0.29 |
| 1 | Carniolan (4) | 8.322 ± 0.29 | 9.535 ± 0.67 |
|  | Italian (1) | 7.771 | 11.369 |
|  | Pol-Line (3) | 9.564 ± 1.04 | 10.870 ± 0.42 |
|  | Russian (8) | 8.997 ± 0.58 | 9.778 ± 0.49 |
|  | Saskatraz (3) | 10.664 ± 0.85 | 9.778 ± 0.75 |
| 2 | Carniolan (4) | 9.053 ± 1.55 | 9.016 ± 0.25 |
|  | Italian (5) | 10.506 ± 0.54 | 10.103 ± 0.55 |
|  | Pol-Line (4) | 8.537 ± 1.04 | 10.776 ± 0.66 |
|  | Russian (4) | 9.881 ± 1.03 | 10.329 ± 0.62 |
|  | Saskatraz (7) | 8.285 ± 0.98 | 10.733 ± 0.35 |
| 3 | Carniolan (8) | 10.219 ± 0.65 | 10.100 ± 0.47 |
|  | Italian (9) | 10.414 ± 0.68 | 10.103 ± 0.41 |
|  | Pol-Line (16) | 10.356 ± 0.46 | 10.458 ± 0.28 |
|  | Russian (6) | 10.023 ± 0.77 | 9.992 ± 0.42 |
|  | Saskatraz (11) | 9.696 ± 0.56 | 9.878 ± 0.31 |
